# Structural Elucidation of Na_2/3_NiO_2_, a Dynamically Stabilized Cathode Phase with Nickel Charge and Sodium Vacancy Ordering

**DOI:** 10.1021/acs.chemmater.5c00084

**Published:** 2025-03-24

**Authors:** James M. A. Steele, Annalena R. Genreith-Schriever, Joshua D. Bocarsly, Liam A. V. Nagle-Cocco, Farheen N. Sayed, Marie Juramy, Christopher A. O’Keefe, Fabio Orlandi, Pascal Manuel, Siân E. Dutton, Clare P. Grey

**Affiliations:** † Yusuf Hamied Department of Chemistry, University of Cambridge, Cambridge CB2 1EW, U.K.; ‡ Cavendish Laboratory, University of Cambridge, JJ Thomson Avenue, Cambridge CB3 0HE, U.K.; § Department of Chemistry and Texas Center for Superconductivity, University of Houston, Houston, Texas 77004, United States; ∥ ISIS Facility, Rutherford Appleton Laboratory, Harwell Campus, Didcot OX11 0QX, U.K.

## Abstract

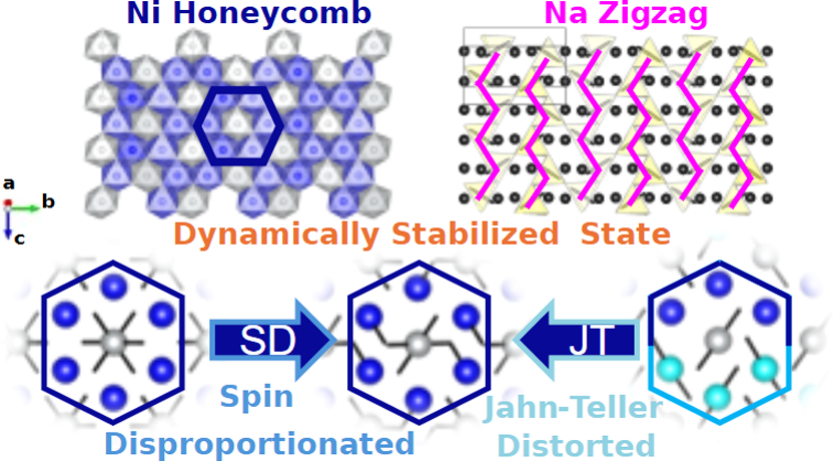

NaNiO_2_ (NNO) has been investigated as a promising sodium-ion battery cathode material, but it is limited by degradation-induced capacity fade. On desodiation, NNO forms multiple phases with large superstructures due in part to Na^+^-ion vacancy ordering; however, their structures are unknown. Here, we report a structural solution to the Na_2/3_NiO_2_ (P^′^3) desodiated phase using combined Rietveld refinement of high-resolution synchrotron X-ray (SXRD) and neutron powder diffraction (NPD) data, magnetic susceptibility, and ^23^Na solid-state nuclear magnetic resonance (ssNMR) spectroscopy. Our experimental results are compared to *ab initio* molecular dynamics (AIMD) simulations, which indicate multiple low-energy structures that are dynamically populated. We observe a combination of competing effects that contribute to the resultant dynamic nature of the structure, including honeycomb ordering of mixed-valence Ni, orbital ordering of Jahn–Teller (JT) distorted Ni^3+^, and zigzag Na^+^/vacancy ordering. Our work provides evidence of multiple contributions to the structures of desodiated Na_2/3_NiO_2_, along with a framework for investigating the other unsolved desodiated structures. This work may also inform our understanding of the Jahn–Teller evolution in other nickel-rich lithium- and sodium-ion cathodes, such as LiNiO_2_.

## Introduction

Na-ion batteries are well-suited to large-scale, low-cost applications. Na is relatively uniformly distributed across the globe, being more than 1000 times more abundant than Li in the Earth’s crust (23,000 ppm vs 20 ppm).^1^ However, Na is larger and heavier than Li, meaning the volumetric/specific capacities (charge per unit volume/mass, respectively) of sodium-ion batteries are inherently lower than those of equivalent lithium-ion battery cathodes (i.e., NaNiO_2_ vs LiNiO_2_). Therefore, optimizing the performance, stability, and reversibility of Na-ion batteries is an important research objective.

Ni-rich cathodes for both Na and Li-ion batteries are the subject of intensive research efforts, with the aim of increasing energy density while decreasing reliance on the more expensive and scarce resource cobalt.^2,3^ For Li-ion batteries, the parent compound LiNiO_2_ has a structure that remains controversial due to open questions about the nature of the Jahn–Teller (JT) distorted *d*^7^ Ni^3+^ cations and possible co-operative JT ordering.^4−8^ The average crystal structure reported from combined refinement of synchrotron X-ray (SXRD) and neutron powder diffraction (NPD) data is rhombohedral , containing no bulk cooperative JT distortion.^9,10^ Computational and experimental studies have put forward evidence of both order–disorder and displacive models for the JT transition, where the term “dynamic stabilization” was used to describe this phenomenon.^11,12^ A complete description of the structure is complicated further by the presence of antisite defects and off-stoichiometry (primarily lithium deficiency), which are difficult to control reproducibly in the synthesis of this material.^13,14^ The complexity of the parent LiNiO_2_ phase adds further complexity to the understanding of the phases that form upon electrochemical cycling, hindering efforts to rationally improve the performance.

In contrast to LiNiO_2_, the pristine Na-analog parent compound (NaNiO_2_) is in many ways a simpler system, as the greater size of the Na^+^ cation prevents the formation of antisite defects, improving reproducibility between synthesized materials and facilitating consistent structural and electrochemical properties.^15^ Furthermore, the larger Na^+^ cation contributes to increased volume changes, and a range of stable, desodiated (Na_1–*x*_NiO_2_) phases are observed during cycling.^15−17^ At room temperature, colinear cooperative ordering of JT-distorted octahedra in NaNiO_2_ results in a monoclinic (*C*2/*m*) structure termed O^′^3, as opposed to the rhombohedral O3 unit cell of the non-JT analog compounds (e.g., NaCrO_2_ [Cr^3+^, *d*^3^], NaCoO_2_ [Co^3+^, *d*^6^]) (Figure S1). Here, the terminology O3 denotes octahedral (O) coordination of the Na^+^ ions, with three TMO_2_ slab layers required to describe the stacking of the unit cell, as per Delmas’ naming convention.^18^ Similarly, P would denote prismatic alkali-ion coordination. Distortion from ideal O/P phases is denoted by a prime marker (^′^), with the number of markers increasing for each new phase in the order of reporting. On heating, NaNiO_2_ undergoes a reversible first-order displacive Jahn–Teller phase transition between 465 and 495 K, resulting in an O3 phase that is isostructural with the Cr/Co analogues and with LiNiO_2_.^19,20^

Sodium-ion battery cathodes, in general, are known to exhibit stable phases throughout their voltage profiles, with ordered arrangements of sodium ions (Na_Na_^*x*^) and sodium-ion vacancies (V_Na_^′^). For example, in Na_1–*x*_CoO_2_, at least two ordered phases are observed (i.e., Na_2/3_CoO_2_ and Na_1/2_CoO_2_).^21,22^ Similarly, the voltage profile of NaNiO_2_ presents a range of characteristic biphasic phase transitions as a function of Na content (Na_*x*_NiO_2_; x ∼ 1, 2/3, 1/2, 2/5, 1/3, and 0.91), spanning both octahedral and prismatic Na coordination environments.^18,23^ This results in the overall transformation of O^′^3 (Na_1_) → P^′^3 (Na_2/3_) → P^′′^3 (N*a*_1/2_) → O^′′^3 (Na_2/5_) → O^′′′^3 (Na_1/3_). The system returns to O^′′′′^3 (Na_∼0.91_) at the end of discharge, as it does not appear to be possible to fully resodiate *via* electrochemical methods.^15−17^ The origins of this complex phase evolution are not well understood but could be expected to be related to the interplay of JT distortions of the *d*^7^ (t_2g_^6^, e_g_^1^) Ni^3+^ ions and Na vacancy and Ni charge ordering. This was highlighted in a recent computational study by Langella *et al.*, in which cooperative Jahn–Teller effects associated with Mn^3+^, combined with changing Na^+^ orderings, were identified as the main driving force for phase transitions present in Na_*x*_MnO_2_ cathodes upon cycling.^24^ Understanding the structural evolution of Na_*x*_NiO_2_ provides an excellent opportunity to study the role of charge, orbital, and vacancy ordering in Ni-rich cathode materials.

Structural solutions for the desodiated Na_*x*_NiO_2_ phases have not been reported, though pattern indexing of XRD data identified lattice parameters and possible space groups for each phase.^15−17,23^ Based on these, the O/P phases were assigned through cell parameters, as prismatic unit cells typically have a greater β (monoclinic) angle than octahedral phases (typically ∼120^°^ vs ∼110°). De Boisse identified small superstructure diffraction peaks and determined supercell space groups and lattice parameters (but not atomic positions) from synchrotron X-ray diffraction data as a function of the parent O^′^3 lattice parameters (cell_O′3_) for NaNiO_2_ (space group = C2/m, *a*_O′3_ = 4.970 Å, *b*_O′3_ = 2.862 Å, *c*_O′3_ = 5.742 Å): P^′^3 (Na_2/3_NiO_2_) = P2_1_/a [*a*_O′3_*3*b*_O′3_*c_O′3_], P^′′^3 (Na_1/2_NiO_2_) = P2_1_/m [*a*_O′3_*2*b*_O′3_*c_O′3_], O^′′^3 (Na_2/5_NiO_2_) = C2/m [*a*_O′3_*5*b*_O′3_*c_O′3_].^25^ A structure for the P^′^3 phase was presented in a density functional theory (DFT) and experimental study of Na_2/3_MnO_2_, which focused on calculations. However, the reported structure obtained after Rietveld refinement of in situ SXRD data results in unphysical Ni–O and Na–O bond lengths.^26^

Here, we report a structure for the first desodiated phase of NaNiO_2_, P^′^3 Na_2/3_NiO_2_, prepared electrochemically. Our structure is consistent with previous studies of the lattice parameters and symmetry,^25,26^ but by using a combined refinement of high-resolution SXRD and NPD data, we are now able to elucidate a complete structural model. We observe both Ni charge and Na vacancy ordering in honeycomb and zigzag arrangements, respectively. Two crystallographically distinct Ni sites are observed, present in a 2:1 ratio, with clear signs of charge disproportionation. Our model is consistent with magnetic susceptibility and solid-state ^23^Na NMR measurements, which further demonstrate that the proposed model additionally describes the local structure. From *ab initio* molecular dynamics (AIMD) simulations, we find frequent transitions between multiple low-lying structures, all of which have Ni charge and Na vacancy orderings, revealing the dynamic nature of the system.

## Experimental Methods

### NaNiO_2_ Synthesis

NaNiO_2_ was synthesized *via* a solid-state route. All reactant powders (NiO, Alfa Aesar Puratronic 99.998%, Na_2_O_2_, Sigma 97%) were mixed and ground manually using an agate pestle and mortar for 15 min within an Ar-filled glovebox, before being pelletized using a pellet press at approximately 5 MPa, then transferred to an alumina crucible. All syntheses were carried out at 700 °C (ramp rate = 3 °C min^–1^, cooling ramp rate set to 10 °C min^–1^ resulting in cooling at ambient rate in the absence of active cooling) for 10 h, under continuous O_2_ flow at a rate of approximately 30 mL min^–1^. Air/moisture exposure was minimized through rapid transfer of the product from the furnace to an Ar-filled glovebox, though it was not possible to eliminate exposure completely.

### Cell Fabrication, Electrochemical Cycling, and *Ex Situ* Sample Preparation

In order to prepare large sample quantities of the desodiated phase suitable for NPD, we used 1 in. diameter Swagelok cells. The synthesized active cathode material (NaNiO_2_) and conductive carbon (Super P) were mixed in a 70:30 ratio for ∼15 min using an agate pestle and mortar, then the resultant powder was added directly to the Swagelok stainless steel current collector. The cells were assembled in the following order, starting from the stainless steel plunger, stainless steel current collector, cathode/carbon mixture, 2 × 1 in. fiber glass spacer, 400 μL electrolyte (1 M NaPF_6_ in propylene carbonate [PC], produced as required to minimize degradation), 15/16 in. Na metal anode, stainless steel current collector, a rigid spring, and stainless steel plunger. The body of the cell was wrapped with Kapton film internally to prevent short-circuiting/degradation. See Figure S2 for more information. The Na metal anodes were produced at the time of use as follows: since the Na metal is stored in mineral oil, the oil was first washed off in a glass vial using heptane, before the metal was rolled to a suitable thickness and punched manually with a 15/16 in. manual punch.

Swagelok cells were cycled using a BioLogic potentiostat, controlled by EC-Lab software, in a temperature-controlled room (at 25 °C), using a charge rate of C/100, followed by a voltage hold at 2.9 V for 48 h to aid in complete conversion to the desired phase. All potentials are referenced vs Na metal anode.

Following electrochemical cycling, samples were retrieved by disassembling the Swagelok cells, scraping the electrode powder from the current collector into a glass jar, and washing with dimethyl carbonate (DMC). The cathode powders were allowed to settle before the DMC (containing any remaining electrolyte and salt) was removed *via* syringe, with any remaining DMC being evaporated under a dynamic vacuum of −1 bar (relative pressure) for 2 h. All disassembly and washing was carried out in an Ar-filled glovebox.

All samples were stored in sealed glass vials in an Ar-filled glovebox until required for measurement to prevent exposure to air/moisture.

### X-ray Powder Diffraction

SXRD experiments were carried out at beamline I-11 at Diamond Light Source, UK.^27^ All powder samples were transferred to 0.5 mm diameter borosilicate glass capillaries and sealed with epoxy glue within an argon-filled glovebox to prevent air/moisture exposure. The samples were measured using the multianalyser crystal (MAC) detectors or Position Sensitive Detectors (PSD) as indicated in the text, at energies/wavelengths of 15 keV (λ = 0.827 Å) as refined against a Si standard. Measurements using the MAC detectors were collected at a step size of 0.001°, and processed through rebinning at 0.010°. All reported measurements were collected at room temperature (approximately 25 °C).

### Neutron Powder Diffraction

Neutron Bragg diffraction measurements were obtained using the wide-angle in a single histogram (WISH) diffractometer at the ISIS Neutron and Muon Source, UK, which provides a very high signal-to-noise ratio in the region where superstructure peaks are expected.^28^ Within a helium-filled glovebox, samples were loaded into 5-mm internal diameter vanadium cans and sealed with indium wire to prevent air/moisture exposure. The cans were loaded onto a sample changer, which was placed within the instrument sample tank under a vacuum. Data were collected across all 5 pairs of instrumental banks (average 2θ of bank pairs 1_10 = 27.0°, 2_9 = 58.33°, 3_8 = 90.00°, 4_7 = 121.66°, and 5_6 = 152.82°), on the sample containing ∼100 mg of Na_2/3_NiO_2_ and ∼43 mg of carbon (the cathode mixture), for approximately 4 h to ensure a suitable signal-to-noise ratio for distinguishing the observed superstructure peaks. All reported measurements were collected at room temperature (approximately 25 °C).

### ISODISTORT + TOPAS-Academic Superstructure Refinements

Rietveld and Pawley refinement of XRD/NPD data was carried out using TOPAS-Academic,^29−31^ with additional input from ISODISTORT as referred to in the Discussion section.^32,33^ For combined refinement of SXRD/NPD data, the data were weighted such that the total contributions of the one XRD pattern and five NPD patterns, collected across the WISH instrument’s 10 (five mirrored sets of two) banks, were equal.^28^

The XRD pattern background was fitted with a Chebyshev polynomial with 38 terms. XRD peak shape contributions from the beam were accounted for using a Thompson-Cox-Hastings pseudo-Voigt peak shape, the parameters of which were refined against a measured Si standard.^34^ Sample contributions to peak shapes were modeled using Lorentzian and Gaussian crystallite size parameters, and Stephens monoclinic strain broadening.^35^

The complex nature of the NPD pattern backgrounds necessitated the use of a user-defined background using the bkg_file() macro in TOPAS-Academic. The background was generated using the automated background function in the WinPLOTR application in the Fullprof software suite,^36,37^ followed by interpolation of these peaks using Python. Instrumental contributions to peak shapes (DIFC, DIFA, ZERO, and tauf_1) in the NPD data were first fit to the measured NaCAlF standard. DIFC, initially 0.0, was later allowed to refine for all but the highest resolution bank, allowing for small differences in sample position within the instrument. The ZERO (which accounts for timing signal differences and finite response times in electrical components of the instrument) and tauf_1 (used in the moderator correction) parameters were kept constant.^38^ These instrumental parameters were incorporated into the TOF Lorentzian and Gaussian crystallite size and strain parameters, in addition to a TOF_2FP_Voigt peak shape.

### Magic-Angle Spinning Solid-State NMR

Samples were center packed into 1.3-mm diameter ZrO_2_ magic-angle spinning (MAS) rotors in an argon-filled glovebox. The rotors were additionally packed with Teflon tape due to low sample quantity. ^23^Na spectra were measured using either a Bruker Biospin Solid-State AV500 (500 MHz, 11.7 T) with 60 kHz MAS and a “H13708 MASDVT500W2 BL1.3 N-P/F-H” probe, or a Bruker AVANCE NEO (400 MHz, 9.4 T) spectrometer, with a “1.3 mm LTMAS H/FXY” probe. ^23^Na spectra were referenced to NaCl as an external reference at 0.0 ppm. Experiments were optimized to enable direct excitation of ^23^Na nuclei (pulse length = π/4), utilizing Hahn-echo and projection magic-angle turning phase-adjusted sideband separation (pj-MATPASS) pulse sequences.^39−41^ For variable temperature measurements, stated values are those of the sample, estimated based on the known relationship between the spin–lattice relaxation time (*T*_1_) and temperature of the ^79^Br spins in a KBr sample (measured in a separate experiment), with intermediate points based on the empirical relationship between measured temperature and temperature calculated at set points.^42^

### Magnetic Measurements

Magnetic property measurements were performed by using a Quantum Design Magnetic Property Measurement System (MPMS3). Within an argon-filled glovebox, the sample of 26.9 mg total mass (18.8 mg active cathode mass) was wrapped in polyethylene film and loaded into polypropylene powder sample capsules. The sample holders were then mounted into a brass sample holder and measured in vibrating sample magnetometer mode.

DC magnetic susceptibility measurements of χ(*T*) = d*M*/d*H* were performed on an *ex situ* sample. In order to study the magnetic behavior and possible magnetic ordering at low temperatures, susceptibility as a function of temperature (on heating) was collected under both zero-field-cooled (ZFC) and field-cooled (FC) conditions, between 1.8 and 300 K, under a constant external field of 100 Oe. Additionally, FC data between 1.8 and 350 K were collected under a field of 20 kOe (Figure S9a)

At all fields, the magnetic susceptibility is in the low field limit where χ(*T*) = d*M*/d*H* ≈ *M*/*H*, and the χ(*T*) measured at 20 kOe was fit between 175 and 350 K using the Curie–Weiss law:
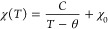


Where *C* is the Curie constant, θ is the Weiss temperature, and χ_0_ is a temperature-independent susceptibility term that accounts for constant diamagnetic or paramagnetic contributions to the signal (which may arise from the sample itself or from the sample holder or electrode additives).

Magnetic susceptibility was also measured as a function of magnetic field strength between 70 and −70 kOe at temperatures of 1.8, 11, 18, 50, and 100 K (Figure S9b).

### DFT, AIMD, and NMR Shift Calculations

DFT calculations were performed with the all-electron CRYSTAL software package using the hybrid functional B3LYP with 20% Fock exchange.^43^ The basis sets proposed by Vilela Oliveira et al. were used on supercells comprising 22 ions.^44^ Geometry optimizations were performed until the energies differed by no more than 10^–6^ eV and the forces by no more than 0.001 eV/Å. A Monkhorst–Pack *k*-point grid of 4 × 4 × 4 was chosen for the geometry optimizations.

Single-point calculations of the energies and the spin density at the nucleus, decisive for the Fermi contact shift, were performed with a finer *k*-point grid of 6 × 6 × 6 *k*-points. Effective magnetic moments were obtained by using the default CRYSTAL projection of the spin densities onto atomic sites. The hyperfine coupling constant and Fermi contact shift were calculated from the nuclear spin density according to Kim et al.,^45^ and scaled to 320 K (while the experiment was nominally conducted at room temperature, frictional heating of the rotor results in a sample temperature of ca. 320 K) using the Curie–Weiss parameters reported in this work.

AIMD simulations were performed according to the generalized gradient approximation (GGA) proposed by Perdew et al.,^46^ and the projector augmented wave method (PAW),^47^ as implemented in the Vienna *ab initio* simulation package (VASP).^48,49^ The plane-wave energy cutoff was set to 500 eV, and a 2 × 2 × 2 Monkhorst–Pack *k*-point grid was used.^50^ Simulations at varying temperatures were performed with the unit cell comprising 22 ions and checked against calculations with a 2 *∼ a*_O′3_× 6 ∼ *b*_O′3_× 2 ∼ *c*_O′3_ supercell comprising 176 ions, yielding nearly identical van Vleck plots of the *Q*_2_, *Q*_3_ ordering parameters. The convergence criterion for the electronic relaxations was set to 10^–6^ eV.

For Ni, the 4*s*^2^3*d*^8^ electrons were treated as valence electrons. To account for the strongly correlated *d* electrons, a rotationally invariant Hubbard *U* parameter of *U*_eff_ = 6 eV was selected, which was used successfully in previous studies of layered oxide cathodes, including the pristine parent material NaNiO_2_.^4,5,20,51^ For oxygen, the 2*s*^2^2*p*^4^ electrons were considered in the valence.

AIMD simulations were performed for the isothermal–isobaric ensemble *(*NpT, constant pressure, particle number, and temperature) at zero pressure. A Langevin thermostat was used with friction coefficients set to 10 ps^–1^.^43−45^ The van Vleck mode analysis of the AIMD snapshot cells was carried out using the Python-based VanVleckCalculator software.^52^ Code is available on GitHub.^53^

## Results

### Synthesis of the P^′^3 Phase

The pristine active cathode material (NaNiO_2_, O^′^3) was synthesized as described in the Methods section, with identity and phase purity confirmed by Rietveld refinement of the literature-reported structure against SXRD data (Figure S3). Prior to the electrochemical synthesis of the P^′^3 phase, complete charge–discharge cycles were measured, showing the expected behavior and allowing for the selection of the appropriate voltage (2.9 V for Na_2/3_NiO_2_, P^′^3) to isolate phase-pure samples (Figure S4a,b).^15^ Because of concerns of reversibility, the samples were always prepared on fresh electrodes, i.e., during the first charge. The phase purity of *ex situ* samples was confirmed by XRD/NPD (Figures S5a–d and S6a,b).

### Structure of the P^′^3 Phase (Na_2/3_NiO_2_)

**Figure 1:**
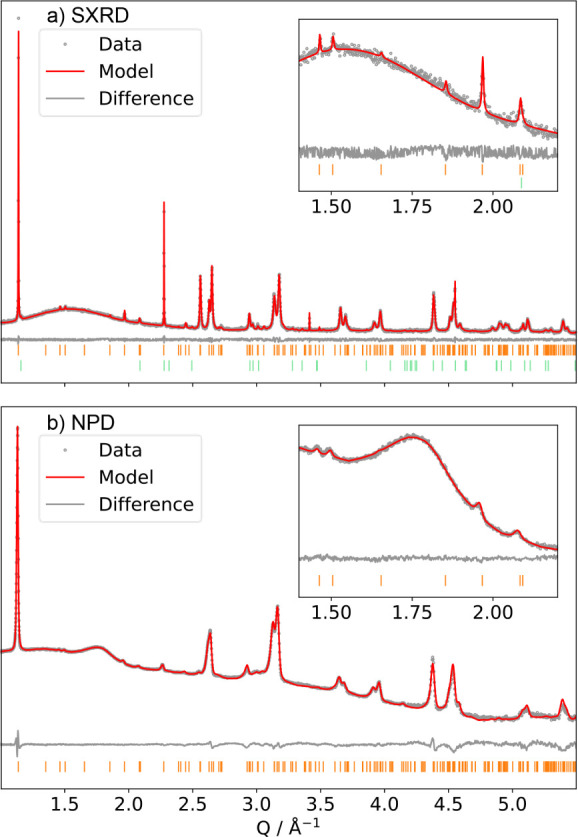
Combined refinement of (a) SXRD and (b) NPD (average 2θ of bank pairs 58.33 ^o^). Tick marks are displayed below for P^′^3 (orange) and the O^′′′′^3 minor impurity (green) in the SXRD sample. Data were collected at room temperature (approximately 25 °C). The square root of intensity is plotted on the *y*-axis for visual clarity. Insets show the superstructure peak region.

From SXRD, the P^′^3 was found to be phase pure (Figure S5c) and, in agreement with the literature, superstructure peaks were observed in the range of 1.40–2.20 Å^–1^ (Figure 1a,b).^25^ NPD collected on the WISH diffractometer shows superstructure peaks in the same *Q* range (Figure S6b). All observed superstructure peaks in both data sets could be indexed to a cell consistent with the previously reported space group (P2_1_/c) and lattice parameters (*a* = 4.972 Å, *b* = 8.589 Å, *c* = 5.739 Å, β = 105.84°) (Figure S7a–d) consistent with the previous report.^25^

ISODISTORT was used to produce candidate structural models for the P^′^3 structure.^33^ As the fully sodiated (O^′^3) phase has octahedrally coordinated Na ions, we started with a hypothetical parent P^′^3 structure (Table ST1) with no superstructure ordering. The lattice parameters of this P^′^3 parent cell were set to values refined by fitting the SXRD pattern, ignoring the superstructure peaks. ISODISTORT was then used to generate a range of symmetry-allowed candidate 1 × 3 × 1 superstructures (*k* point: 0, 1/3, 0) with space groups that would be consistent with the observed superstructure peaks (Table ST2). Of these candidate structures, the best fit with the lowest *R*_wp_ was found using a structure with space group P2_1_/c (irreducible representation LD2 k2t2, with order parameter direction P1) (Table ST3). By comparison to the original (O^′^3, C2/m) and hypothetical (P^′^3, C2/m) structures, which contain single Na, Ni, and O sites, the lower symmetry structure (P^′^3, *P*2_1_/c) with ∼ *a*_O′3_×3 ∼ *b*_O′3_ ×∼ *c*_O′3_, (approximate due to refinement of lattice parameters in hypothetical parent to SXRD data) contains: 2 Ni sites (Ni_(1)_ = 2*a,* Ni_(2)_ = 4*e*) generating honeycomb ordering, 3 possible Na sites (all 4*e*), which would be expected to have 1/3 occupancy each if all occupied, and 3 O sites (all 4*e*), allowing for Ni orbital (JT), charge, and Na vacancy ordering. Refinement of Na site occupancies indicates that a single Na (Na_(3)_ 4*e*) site is fully occupied, with the other sites (Na_(1)_, Na_(2)_) vacant, giving a composition of Na_2/3_NiO_2_, consistent with literature reports and our electrochemistry (Figure S4a,b).^16,17^ In subsequent refinements, the occupancies of the Na_(3)_ and Na_(1/2)_ sites (4*e*) were fixed to 1 and 0, respectively. Further refinement of the allowed atomic coordinates for Ni and O and the occupied Na_(3)_ site enabled a good fit to the data, with all superstructure peaks fit well in both the SXRD and neutron diffraction (*R*_wp_ = 1.473).

**Figure 2:**
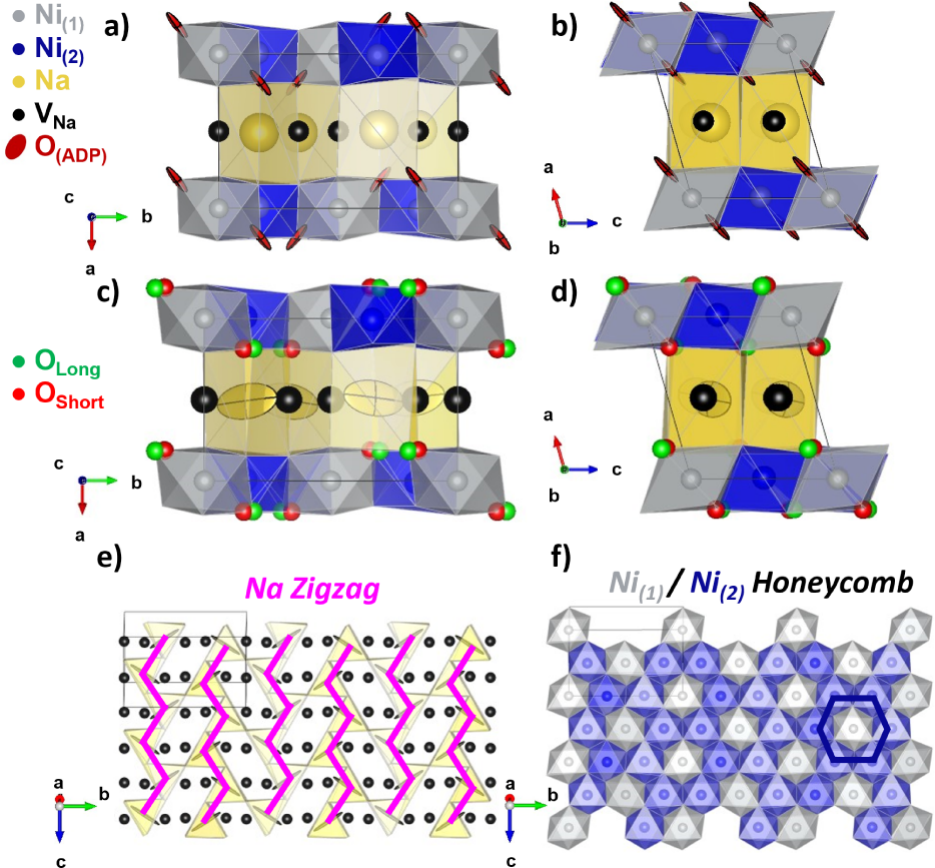
Structural model for the P^′^3 phase as determined from combined refinement of SXRD and NPD data. The structural model with anisotropic ADPs for the O_(3)_ site is shown along the (a) c and (b) b directions. The structural model with anisotropic ADPs for the Na_(1)_ site, and split O_(3)_-site is shown along the (c) c and (b) b directions. (e) Na/vacancy zigzag ordering of edge-sharing occupied Na trigonal prismatic sites (in yellow) in the Na-layer (pink zigzags overlaid for visual clarity to show the repeating zigzag motif). (f) Honeycomb ordering of the two Ni sites on the triangular Ni lattice. In (e)/(f) 1 × 3 × 3 unit cells are shown, while the unit cell itself is denoted by the “black box outline”.

In the refined structure, we find large isothermal (*B*_iso_) parameters for the O_(2)_ and Na_(3)_ sites, with all other thermal parameters refining to 0.498–1.155 Å^2^. Adding anisotropic atomic displacement parameters (ADPs) for the O_(2)_ site results in a large, thin displacement ellipsoid along the direction of the Ni_(1)_–O_(2)_ bond, suggesting significant vibrations or disorder along the JT axes. Alternatively, we can model O_(2)_ using a split site model with one long and one short Ni_(1)_–O_(2)_ bond, which refine to 2.172(24) and 1.914(26) Å, respectively. When we model the Na_(3)_ site with anisotropic thermal parameters, these manifest as ellipsoids pointing directly through the large prismatic faces of the prismatically coordinated Na, directly into a vacancy (known to be a facile pathway for Na mobility).^54^ The refined structure is displayed in Figure 2a–f (full structural details are presented in Table ST4).

Our structural model contains edge-sharing triangular prisms occupied by Na^+^ ions (termed “herringbone” patterning by Wang *et al.* but more widely referred to as “zigzag”*)*,^55^ and a honeycomb ordering of the Ni sites, with the occupied Na sites broadly correlated with the more contracted Ni_(2)_O_6_ octahedra in adjacent layers (Figure 2e,f). The two Ni sites have dramatically different bond lengths, with average Ni–O bond lengths for the honeycomb Ni_(1)_ and Ni_(2)_ sites of 1.985(15) and 1.93(8) Å, respectively. This suggests that the two Ni sites have different ionic charges, with the Ni_(1)_ sites having a lower oxidation state relative to Ni_(2)_. We note that Ni–O bonds in nickelates are known to have covalent character; i.e., charge ordering is expected to be reflected both in the Ni and in the O lattices. For the sake of clarity, we will refer to charges in terms of formal ionic charges (assuming ionic bonds) as the formal charges directly correlate with the Ni spin states. The bond valence sum (BVS) method was used to calculate the nominal charge of the two Ni sites. We considered a range of ideal bond length (*R*_0_) values, finding that the most suitable was Ni^4+^–O^2–^ (*R*_0,Ni4+_ = 1.734 Å, *B*_0,Ni4+_ = 0.335 Å). We suspect that this is due to the nonuniform Ni–O bond length distribution caused by JT effects (see S-5 in Supporting Information for more information). We obtain values of Ni^3.07+^ and Ni^3.43+^ for Ni_(1)_ and Ni_(2)_, respectively, giving an average charge of Ni^3.31+^. This is close to the expected value of Ni^3.33+^ for Na_2/3_NiO_2_.

### ^23^Na ssNMR

**Figure 3:**
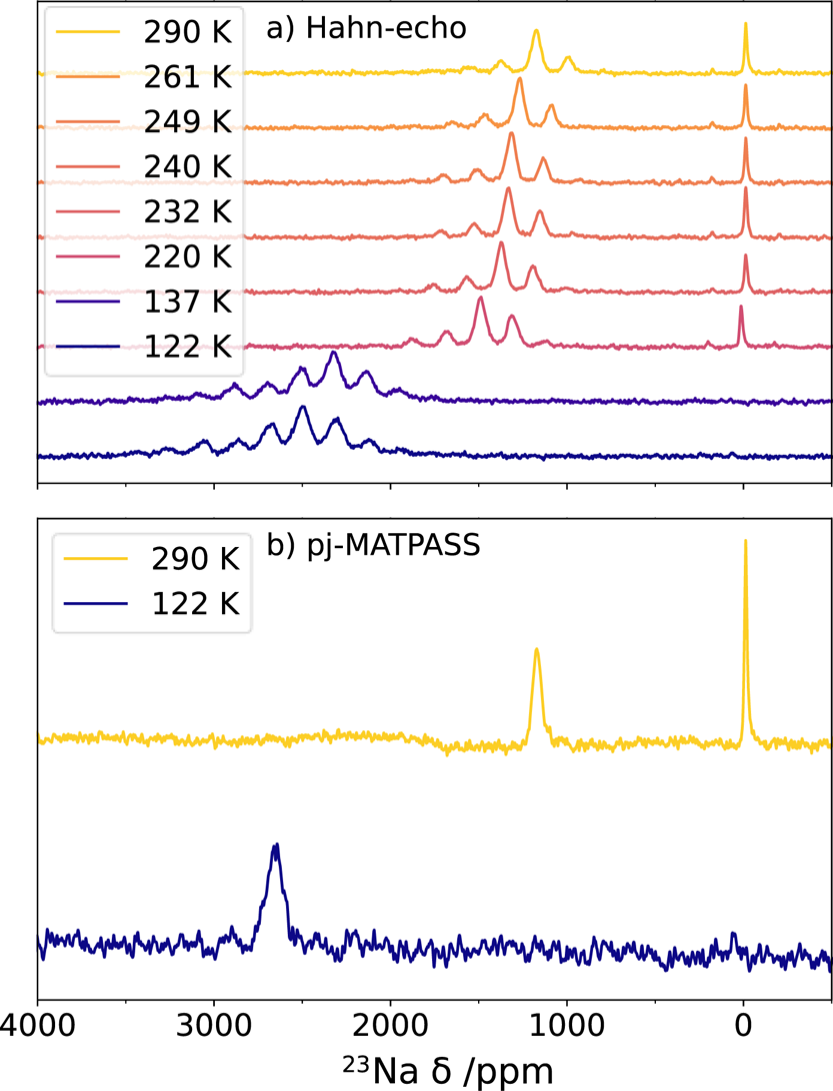
Variable temperature ^23^Na MAS ssNMR: (a) Hahn-echo and (b) pj-MATPASS spectra of the desodiated P^′^3 phase, acquired from 122 K to 290 K with 20 kHz MAS. Measured at 400 MHz (9.4 T) field strength. The offset frequency was moved with temperature to optimize the signal intensity of the higher frequency resonance; the ∼0 ppm diamagnetic peak was consequently no longer observed at low temperatures at the offset frequencies shown in these spectra.

Variable-temperature ^23^Na solid-state magic angle spinning NMR (MAS ssNMR) of ^23^Na was performed on the P3 phase (Figure 3a,b). A broad, high-frequency resonance with a series of spinning sidebands is observed in the Hahn-echo experiment (Figure 3a). The peaks shift further to higher frequencies with decreasing temperature. The large shift is ascribed to a hyperfine (Fermi contact) interaction between the ^23^Na nuclei and the unpaired electrons on the Ni^3+^ ions,^56^ with the increased shift upon decreasing temperature resulting from an increase in magnetic susceptibility (strictly, the time-averaged value of the magnetic moment) with temperature.^57^ A broadening of the spinning sideband manifold seen on cooling can similarly be ascribed to the increase in magnetic susceptibility, in possible combination with decreasing Na^+^-ion mobility (this is currently under investigation but beyond the scope of the present work). We also observe a resonance at ∼0 ppm, which we attribute to diamagnetic impurities such as NaF, NaHCO_3_, and Na_2_CO_3_ from the as-synthesized material and from degradation of propylene carbonate electrolyte.

In the pj-MATPASS experiments—used to separate the isotropic resonance from its spinning sidebands (Figure 3b)—we observe a single isotropic paramagnetic resonance from the P^′^3 phase at room temperature (∼1086 ppm at 290 K), down to the lowest measured temperature (∼2650 ppm at 122 K). This is consistent with the single Na environment in the vacancy-ordered structure refined from the SXRD and NPD data. This resonance is observed at a noticeably lower shift in comparison to that previously reported for the pristine O^′^3 NaNiO_2_ material (∼1086 ppm vs 1460 ppm; for comparison of P^′^3 and O^′^3 see Figure S5e).^20^ We ascribe this to the decreased hyperfine shift, resulting from the oxidation of 1/3 of the Ni^3+^ in the Na_2/3_NiO_2_ phase to diamagnetic Ni^4+^ ions.

### Magnetic Susceptibility and Curie–Weiss Fitting

The magnetic susceptibility of the P^′^3 phase shows paramagnetic behavior at high temperatures, *T* > 100 K. On further cooling, the ZFC and FC susceptibility diverge, and a cusp in the ZFC susceptibility is observed at *T* ≈ 25 K. The low-temperature magnetic properties are tentatively ascribed to either long-range magnetic ordering or spin freezing (spin-glass-like behavior) (Figure S9a). Fitting to the Curie–Weiss law (between 175 K and 350 K) yields Curie Constant (*C*) = 0.251(1) emu K mol^–1^, Weiss temperature (_CW_) = −0.2(7) K, and χ_0_ = −5.5(3) × 10^–5^ emu mol^–1^ Oe^–1^. This Curie constant corresponds to an effective magnetic moment (μ_eff_) of 1.416(4) μ_B_, which is between the expected moments for Ni^3+^ (*S* = 1/2, μ_eff_ = 1.73 μ_B_) and Ni^4+^ (*S* = 0, μ_eff_ = 0 μ_B_) (S-7 in Supporting Information). The Weiss temperature is significantly smaller than what might be expected from the onset of magnetic correlations around 100 K, possibly due to competing ferromagnetic (FM) and antiferromagnetic (AFM) interactions with similar magnitudes leading to a mean field Weiss temperature around zero. The magnetic susceptibility has similarities to that of pristine NaNiO_2_, which forms a long-range order state at 23 K with in-plane FM and interlayer AFM ordering,^58,59^ albeit with a significantly reduced Weiss temperature (−0.2(7) K in Na_2/3_NiO_2_ vs 36 K in NaNiO_2_).^60^

### DFT Calculations and AIMD Simulations

To explore the energetics of charge and cation ordering, single-point DFT calculations of the structure corefined from X-ray and neutron diffraction data were first performed, fixing the atomic positions at the refined values. The electronic structure of the material was found to have mixed valence character, with 1/3 of the Ni ions having a spin of *S*(Ni_(1)_, gray) = 0.47 and 2/3 of the Ni ions having a spin of *S*(Ni_(2)_, blue) = 0.29 (Figure 2f).

**Figure 4:**
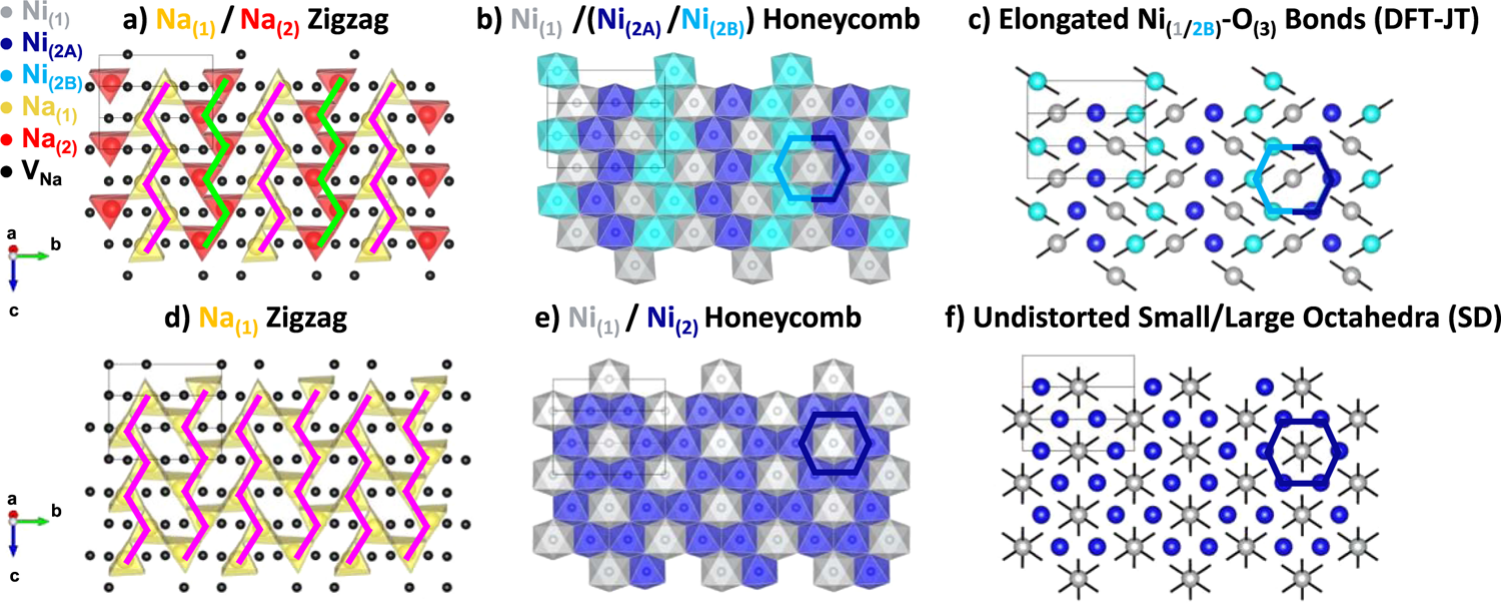
(a–c) Jahn–Teller (DFT-JT) and (d–f) spin disproportionated (SD) structural models for the P^′^3 phase. Na^+^/vacancy zigzag ordering in the Na-layer (pink/green zigzags overlaid) is present in both models (a, d). Honeycomb (or equivalent) ordering of Ni sites on the triangular Ni lattice is also present in both models (b, e). Note that in the DFT-JT model, while there are 3 crystallographic Ni sites, Ni_(1)_ (grey), Ni_(2A)_ (dark blue)/Ni_(2B)_ (light blue), their NiO_6_ environments are essentially identical; thus, they are equivalent for the purpose of charge ordering. Ni–O coordination within the unit cell (cutoff for long bonds drawn = 2.0 Å) differs between the two models (c, f). In these “ball and stick” style figures, the atoms are enlarged for visual clarity. In all panels, 1 × 3 × 3 unit cells are shown, while the unit cell itself is denoted by the “black box outline”.

**Figure 5:**
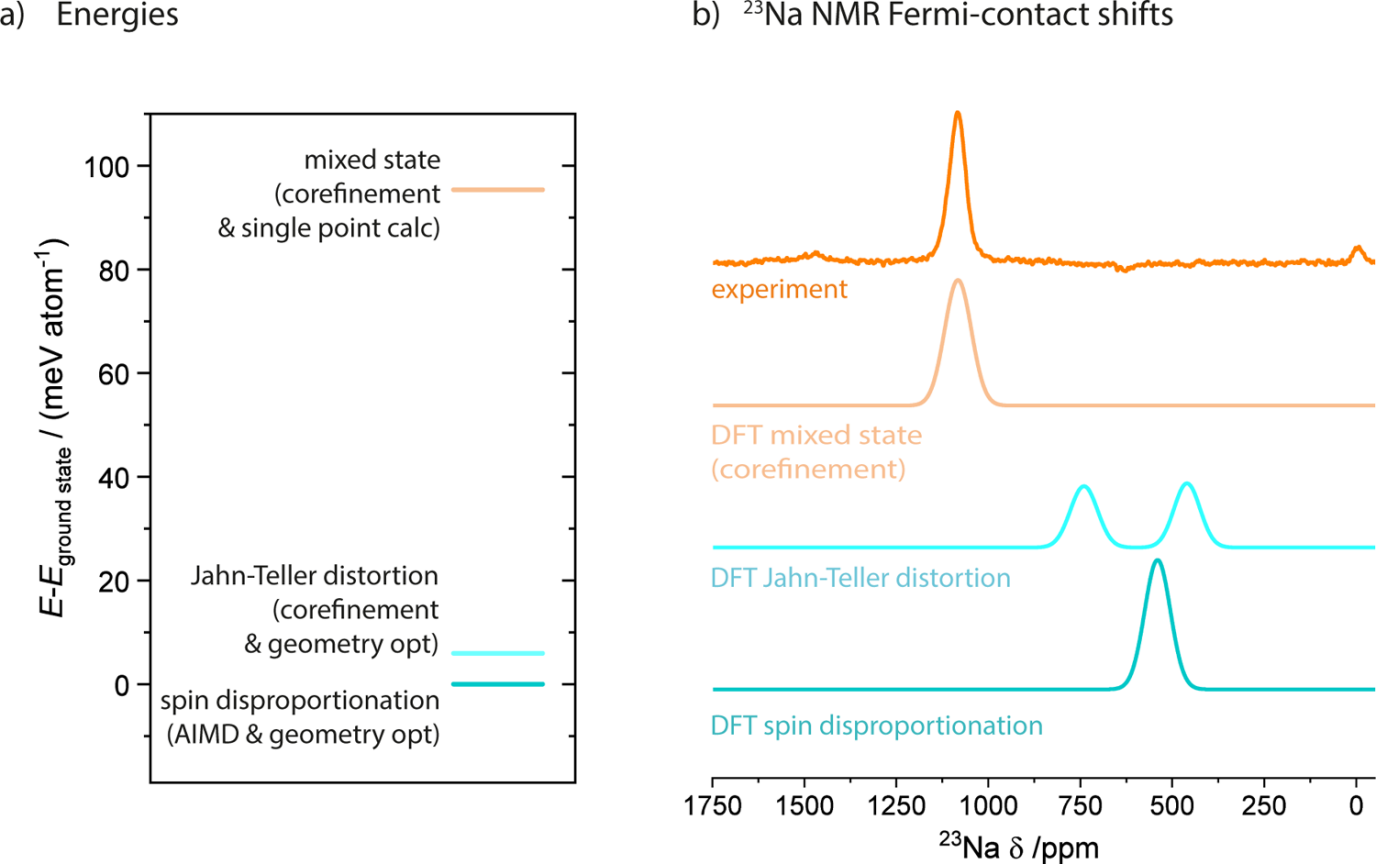
(a) Energetics of the structural models of the P^′^3 phase shown in Figure 4 as obtained from hybrid functional DFT calculations. The energy states are labeled with their respective Ni-O_6_ coordination environments as highlighted in Figure 4c/f. (b) Predicted ^23^Na Fermi contact shifts for the three structural models. Both the spin-disproportionated (SD) ground state and the Jahn–Teller distorted (DFT-JT) state with a similar energy are predicted to exhibit resonances at significantly smaller shifts than the experimental spectrum. The predicted resonance of the experimental SXRD/NPD corefined structure shows excellent agreement with the experimental spectrum.

When the atomic positions were allowed to optimize from the corefined structure (allowing the system to change symmetry in the course of the optimization), the structure relaxed into a lower symmetry Pc structure (Figure 4a–c). In this structure, the Ni_(2)_ (4*e*) site of the corefinement structure was observed to split into Ni_(2A)_ (blue) and Ni_(2B)_ (light blue) (2*a*) sites. Both Ni_(2)_ sites have spins of 0.29. However, only the Ni_(2B)_ (light blue) site is JT distorted. The Ni_(1)_ site is also found to have a JT distortion but has spin *S* = 0.47. The average nominal Ni charge state calculated *via* the BVS method for this model was Ni^3.27+^, with Ni_(1)_ (gray), Ni_(2A)_ (blue), and Ni_(2B)_ (light blue) sites having charges of Ni^3.73+^, Ni^3.04+^, and Ni^3.06+^ respectively (Figure 4b). The structures obtained by DFT are compared in Figure 4 (experimental and calculated structures are all compared in Figure S10); both the honeycomb ordering of Ni and the zigzag Na vacancy ordering are retained from the combined refinement structure in the relaxed cells. However, the relative oxidation of the Ni_(1)_:Ni_(2)_ sites is reversed in the two structures. The energy of this geometry-optimized structure was found to be 89 meV/atom lower than the energy of the corefined structure without geometry optimization (Figure 5a).

To ensure that the overall minimum-energy configuration was found and to check that the optimization did not converge to a local minimum in the energy landscape, AIMD simulations were performed at finite temperatures, allowing the system to overcome energy barriers on the scale of the thermal energy. At low temperatures (*T* < 300 K), the AIMD trajectories show undistorted NiO_6_ octahedra of two different sizes and spin states, with 1/3 of the octahedra being large with a spin of *S*(Ni_(1)_) = 0.84, and 2/3 of the octahedra being small with a spin of S(Ni_(2)_, blue) = 0.0 (Figure 4f). The average nominal Ni charge state calculated *via* the BVS method was Ni^3.22+^, with Ni_(1)_ (gray) and Ni_(2)_ (blue) sites having charges of Ni^2.31+^ and Ni^3.67+^, respectively (Figure 4e). The Na ions retain the zigzag vacancy ordering throughout the AIMD run, with no Na^+^ hopping observed, likely due to the relatively short time frame (Figure 4d). No other symmetry lowering was observed, with the space group and number of Ni/Na sites preserved from the corefined structure. The Ni–O coordination environments and spins of all models are explored in S-8 (Figure S10).

At around room temperature, the AIMD trajectories exhibit a highly dynamic structure oscillating between the spin-disproportionated and JT-distorted states, reflective of the structure obtained *via* combined refinement of diffraction data (Figure S13). A van Vleck analysis of the AIMD trajectories (Figure S12) shows circular clusters of data points at the origin at low temperatures, confirming the undistorted nature of the spin-disproportionated octahedra.^52^ With increasing temperature, the distribution of the data points becomes more triangular, illustrating a tendency of the system to become dynamically JT-elongated (equally in the 3 possible dimensions), which may be considered as phonon-induced soft-JT modes.^11,61^ The highest density of data points, however, is still centered around the pole of the van Vleck plot; i.e., while there are more JT characteristics in the vibrations, the system is still mostly undistorted, stemming both from undistorted spin-disproportionated states and the high-temperature displacive phase of the JT-distorted ground state. Note that when snapshots of the dynamic trajectories at room temperature were geometry optimized (corresponding to 0 K), the snapshots relaxed to the spin-disproportionated state (Figure 4d–f). The spin-disproportionated state was found to be the overall lowest energy configuration, being 5 meV/atom more favorable than the Jahn–Teller distorted state (Figure 5a).

^23^Na NMR Fermi-contact shift calculations were performed to help distinguish between the three structures (experimental SXRD/NPD combined refinement, DFT geometry optimization of the experimental structure [DFT-JT], and AIMD snapshot with geometry optimization at 0 K [spin disproportionation, SD]). The computed values were then compared to the observed ^23^Na room temperature isotropic resonance at 1080 ppm. The ground-state spin-disproportionated structure is predicted to have a ^23^Na NMR resonance at *∼*540 ppm (Figure 5b), while the DFT-JT structure is expected to exhibit resonances at 460 and 740 ppm, owing to the presence of two Na sites in this structure. Both 0 K geometry-optimized structures underestimate the shift of the experimental resonance at 1080 ppm. For the experimental structure, without geometry optimization, a shift of 1078 ppm was predicted, in excellent agreement with the experimentally observed hyperfine shift. To validate this further, shifts were also predicted for the structure corefined under the constraint of split O-site occupancies. The Fermi contact shifts predicted for the split-site structure are very similar to those predicted for the original experimental structure, exhibiting Fermi contact shifts at *∼*1040 and 1110 ppm; two separate resonances may not be resolved, and they may merge to form a broad peak given the large experimental peak line widths.

## Discussion

The results of experimental studies and simulations provide a number of possible structures for P^′^3 Na_2/3_NiO_2_. All have honeycomb ordering of Ni charges and zigzag ordering of Na^+^ vacancies. DFT studies have predicted similar zigzag vacancy ordering as the ground state of Na_2/3_CrO_2_, though Na^+^ is octahedrally coordinated (O^′^3) in this system.^62^ However, our observed combination of honeycomb transition metal ordering and zigzag Na^+^ vacancy ordering in a prismatically coordinated (P^′^3) system is also observed in the mixed transition metal compound Na_2/3_Cu_1/3_Mn_2/3_O_2_.^55^ Here, the orderings are reported to minimize both intralayer Na–Na repulsions (by preventing any high-energy face-sharing Na prisms) and interlayer repulsions by locating the occupied Na sites further from the Jahn–Teller distorted CuO_6_ octahedra. We anticipate similar stabilization of the structure in Na_2/3_NiO_2_, though the reduced charge difference between the Ni sites compared to Cu^2+^/Mn^4+^ decreases the importance of this effect.

The key difference between the corefined structure and the low-energy structures obtained *via* DFT/AIMD is the oxide-ion positions. To check that the initial refinement had not identified a local minimum, the energy-minimized structures obtained from DFT were used as starting structures in combined SXRD and NPD analysis. These starting models resulted in calculated patterns that were good visual fits to the data, including accounting for the superstructure peaks, which are constant across all models (SD *R*_wp_ = 2.929, JT *R*_wp_ = 2.870). However, when the atomic coordinates were allowed to refine, the structure returned to the original structure with Ni_(1)_ and Ni_(2)_ with nominal charges of Ni^3+^ and Ni^3.5+^, respectively. This suggests that neither of the simulated structures (JT distorted or spin-disproportionated) on their own represents a good description of the average room-temperature structure.

Magnetic measurements did not allow us to distinguish between the three structures identified. Calculating the expected effective moments (using eq. SE3) for the three structures produces nearly identical values: 1.41 for the JT-distorted structure, 1.43 μ_B_ for the spin-disproportionated structure, and 1.41 μ_B_ for the corefined structure. These are all in excellent agreement with the effective moment determined from SQUID measurements (1.416[4] μ_B_) (see Section S-7 in Supporting Information).

While the ^23^Na NMR supports the corefined structure, as the calculated shift of this structure matches the experimental data well, its high energy (95 meV/atom above the ground state based on hybrid functional DFT calculations) relative to the spin-disproportioned ground state is sufficiently large that one would not expect it to be accessible at room temperature. While kinetically stabilized phases are known to form during electrochemical deintercalation, the similarities in the three structures, with only subtle changes in O ion coordination rather than significant changes in the underlying structure, make kinetic stabilization unlikely here. We now consider how to reconcile these results.

The two structures identified to be the lowest energy by DFT, the spin-disproportionated (SD) ground-state structure and the lowest-energy excited-state JT-distorted structure (DFT-JT), are nearly degenerate, with an energy difference of only 5 meV/atom. Due to the thermal energy of the system at finite temperatures, both states are expected to be occupied, and transitions between the states are expected to occur, maximizing the system’s entropy through a large number of possible local configurations. This fluctuating behavior is reflected in the AIMD trajectories, which, at low temperatures, mostly exhibit spin-disproportionated characteristics, with the JT contributions increasing with increasing temperature. This can be observed by visually inspecting the AIMD trajectories (Figure S13) and quantified with a van Vleck analysis of the distortion modes (Figure S12).^52^ This analysis allows for quantification of distortion in octahedra. The bond length distortions associated with JT distortion are typically quantified *via* the *Q*_2_ (two short, two “undistorted”, and two long bonds) and *Q*_3_ (“JT elongation”) modes. At low temperatures, the trajectories show a circular distribution of *Q*_2_/*Q*_3_ ordering parameters around the pole of the plot, which is typical of undistorted octahedra. With increasing temperature, the distribution becomes increasingly triangular, resembling that of the high-temperature displacive phase of stoichiometric NaNiO_2_, which, at room temperature, is colinearly JT-distorted.^20^ This suggests that an effect of desodiation is the disruption of the co-operative JT distortion, similar to that of increasing temperature in the stoichiometric parent compound. The resultant phase is dynamically stabilized *via* fluctuations between a configuration with disproportionated octahedra (SD) and a configuration resembling the displacive high-temperature phase in stoichiometric NaNiO_2_. This has been discussed by Radin et al. in the context of Jahn–Teller active layered materials, including NaNiO_2_.^11^

The question then arises of how the predicted structure at room temperature, which oscillates on the picosecond time scale between the nearly degenerate spin-disproportionated and JT-distorted (displacive) structures, would be observed in our experiments. In both diffraction and ssNMR, a time-averaged structure of the two states would be observed. Closer inspection of the corefined structure demonstrates features in the Ni coordination characteristic of dynamic fluctuations between the two structures. The dynamic nature of the structure is reflected in the O_(3)_ positions, which can be modeled either by a split-O site or highly anisotropic ADPs. The parent material, NaNiO_2_, and related layered Ni^3+^ containing layered oxides typically exhibit JT-elongation. In LiNiO_2_, AIMD simulations and experimental observations have demonstrated that the transition (reorienting the long O–Ni–O bond axes) occurs through a transition state with two long axes and one short axis (i.e., four long Ni–O bonds and two short Ni–O bonds).^12^ The Ni_(1)_ sites observed in the corefined structure with two long axes and one short axis are therefore consistent with dynamic fluctuations between the spin-disproportionated and JT-distorted (displacive) structures.

Based on our combined experimental and computational study, we therefore conclude that the structure of Na_2/3_NiO_2_ is dynamically fluctuating between two near-degenerate states which, when time-averaged, is best described by the corefined long-range structure obtained by diffraction methods. It may be possible to experimentally explore the dynamics *via* probes such as inelastic neutron scattering, resonant inelastic X-ray scattering, or muon spin-resonance spectroscopy. However, these are beyond the scope of this study.

Charge ordering in mixed-valence Ni systems is observed in Sm_9_Ni_9_O_22_ (SmNiO_2.44_) prepared from the topotactic reduction of SmNiO_3_ perovskite.^63^ The reduced structure Sm_9_Ni_9_O_22_ contains square planar Ni^+^ and Ni^3+^ in square pyramidal and octahedral coordination environments. The structure of Na_2/3_NiO_2_ is unique within the electrochemically produced layered transition metal Na_2/3_MO_2_ oxides. For M = V and Ti (*x* ∼ 0.68), both form O^′^3 phases with octahedrally coordinated Na. For M = Co, a P2 phase is experimentally observed.^21^ For M = Cr an O^′^3 phase has been predicted,^64^ though it is yet to be reported experimentally.^65^ The key features of Na_2/3_NiO_2_, honeycomb ordering of Ni and zigzag Na ordering, are observed in mixed-metal Na_2/3_Cu_1/3_Mn_2/3_O_2_. We hypothesize that it is the distinct charges of the two Ni sites which stabilize the structure. Both of these P^′^3 phases contain JT active ions (Ni^3+^/Cu^2+^), while the related phase Na_2/3_Mg_1/3_Mn_2/3_O_2_ that adopts a monoclinic Cm structure does not.

## Conclusions

We report the structure of the first desodiated phase of NaNiO_2_: Na_2/3_NiO_2_ (P^′^3), providing a framework to solve the structures of the remaining desodiated phases. Through the combination of experiment and simulations, the structure observed experimentally is found to be best described *via* the time average of dynamic fluctuations between two near-degenerate states. We find that Na_2/3_NiO_2_ has two different Ni environments with distinct nominal charges arranged in a honeycomb ordering, with Na forming a zigzag ordering, accounting for the experimentally observed superstructure reflections. Similar structural motifs have been observed in the bimetallic layered oxide Na_2/3_Cu_1/3_Mn_2/3_O_2_, which contains JT-distorted Cu^2+^ and Mn^4+^ ions. The combined experimental and computational approach based on SXRD/NPD, ^23^Na ssNMR spectroscopy, AIMD simulations, and DFT calculations has proven a powerful tool to characterize the complex dynamic nature of the room-temperature phase of Na_2/3_NiO_2_ and promises to shed light on vibrationally complex/dynamically stabilized materials more generally. Only through a complete understanding of the parent system will it become possible to rationally design next-generation Na-ion cathode materials through the targeted incorporation of TM dopants to disrupt the cooperative effects of TM-orbital and Na-vacancy ordering.

## Supporting Information Available

Description of Jahn–Teller distortions in the parent compound NaNiO_2_, details on the large diameter Swagelok cells, confirmations of sample identities and phase purities, initial structural model for P^′^3, bond valence sum method calculations, details of the Na_2/3_NiO_2_ combined refinement, magnetic characterization and Curie–Weiss fitting, Ni–O coordination environments and Ni spins in the structural models, and a van Vleck analysis of the AIMD trajectories (PDF)
