# Supplementary material for: Structural Elucidation of Na2/3NiO2, a Dynamically Stabilized Cathode Phase with Nickel Charge and Sodium Vacancy Ordering
Source: Chem Mater. 2025 Mar 24;37(7):2581–91. doi: 10.1021/acs.chemmater.5c00084 (PMC11983708; doi:10.1021/acs.chemmater.5c00084)
Supplement: Supplementary file 1 — cm5c00084_si_001.pdf [file cm5c00084_si_001.pdf]

Supplementary Information: Structural Elucidation of $Na_{2/3}NiO_2$, A Dynamically Stabilised Cathode Phase with Nickel Charge- and Sodium Vacancy- Ordering.

James M. A. Steele,[1, 2] Annalena R. Genreith-Schriever,[1] Joshua D. Bocarsly,[3] Liam A. V. Nagle-Cocco,[2,4] Farheen N. Sayed,[1] Marie Juramy,[1] Christopher A. O'Keefe,[1] Fabio Orlandi,[5] Pascal Manuel,[5] Siân E. Dutton [2] & Clare P. Grey [1*]*

[1] Yusuf Hamied Department of Chemistry, University of Cambridge, Cambridge, CB2 1EW, UK

[2] Cavendish Laboratory, University of Cambridge, JJ Thomson Avenue, Cambridge, CB3 0HE, UK

[3] Department of Chemistry and Texas Center for Superconductivity, University of Houston, Houston, TX 77004, USA

[4] Current address: Stanford Synchrotron Radiation Lightsource, SLAC National Accelerator Laboratory, Menlo Park, CA 94025, USA

[5] ISIS Facility, Rutherford Appleton Laboratory, Harwell Campus, Didcot OX11 0QX, UK

[*] Email: sed33@cam.ac.uk, cpg27@cam.ac.uk

Contents

S-1: Jahn-Teller Distortion in the Parent Compound $NaNiO_2$ 3
S-2: Large Diameter Swagelok Cells for Synthesis of High Quantity *Ex Situ* Sample 4
S-3: Confirmation of Sample Identities & Phase Purity 5
S-4: Initial Structural Model for P/3 9
S-5: Bond Valence Sum (BVS) Method Calculations 12
S-6: Details of the $Na_{2/3}NiO_2$ Combined Refinement 13
S-7: Magnetic Characterisation and Curie-Weiss Fitting 19
S-8: Ni-O Coordination Environments and Ni Spins in the Structural Models 21
S-9: A van Vleck Analysis of AIMD Trajectories 22
References: 25

S-1: Jahn-Teller Distortion in the Parent Compound $NaNiO_2$

Fully sodiated NNO has been investigated extensively from a magnetic perspective due to its triangular magnetic lattice, with a well-studied low-temperature antiferromagnetic ordering (Néel temperature T_N = 20 K), resulting from antiferromagnetic coupling between the individually ferromagnetic transition metal oxide layers (NiO_2 "slabs"). In addition, for $T < 460$ K, ferrodistortive orbital ordering arises due to the cooperative JT effect in d^7 Ni^{3+}, whereby the NiO_6 octahedra become axially elongated and equatorially compressed, with two long and four short Ni-O bonds (Figure 4). Elongation in the z-direction (with respect to conventional cartesian axes for the d-orbitals), removes the orbital degeneracy by lowering electron-electron repulsive interactions between the central metal (Ni^{3+}) ion, and the six bonded O^{2-} ions, which are all situated directly along these axes. This distortion lowers the energy of the occupied d_{z^2}, d_{xz} and d_{yz} orbitals, stabilising the structure. The cooperative ordering of JT-distorted octahedra results in the monoclinic ($C2/m$) O′3 unit cell of NNO, as opposed to the rhombohedral ($R\bar{3}m$) O3 unit cell of non-JT analogue compounds (*i.e.* $NaCoO_2$ [Co^{3+}, d^6]).[1–4]

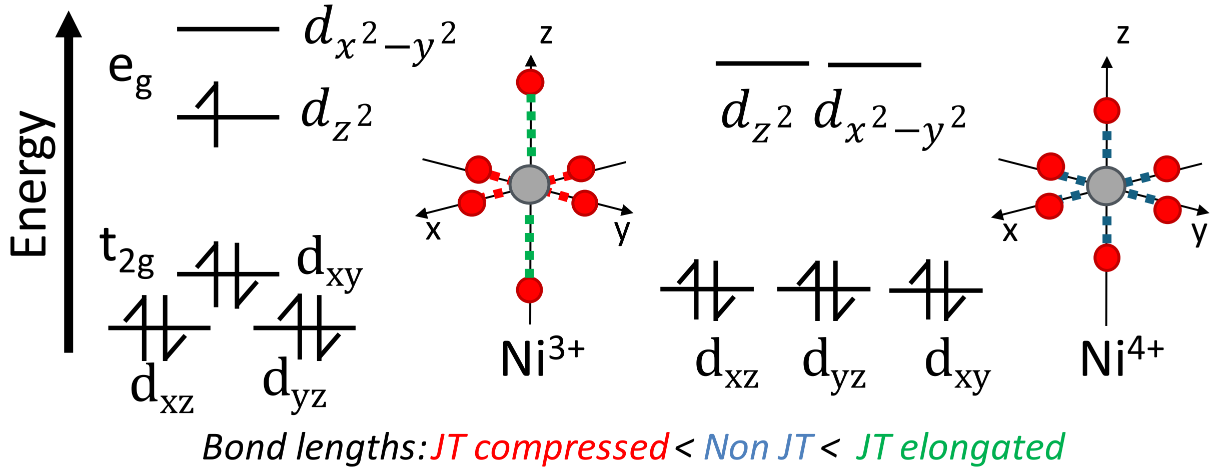

Figure S1: Electronic configurations, and resultant JT effect of JT active d^7 Ni^{3+} (left), and JT inactive d^6 Ni^{4+} (right).

S-2: Large Diameter Swagelok Cells for Synthesis of High Quantity *Ex Situ* Sample

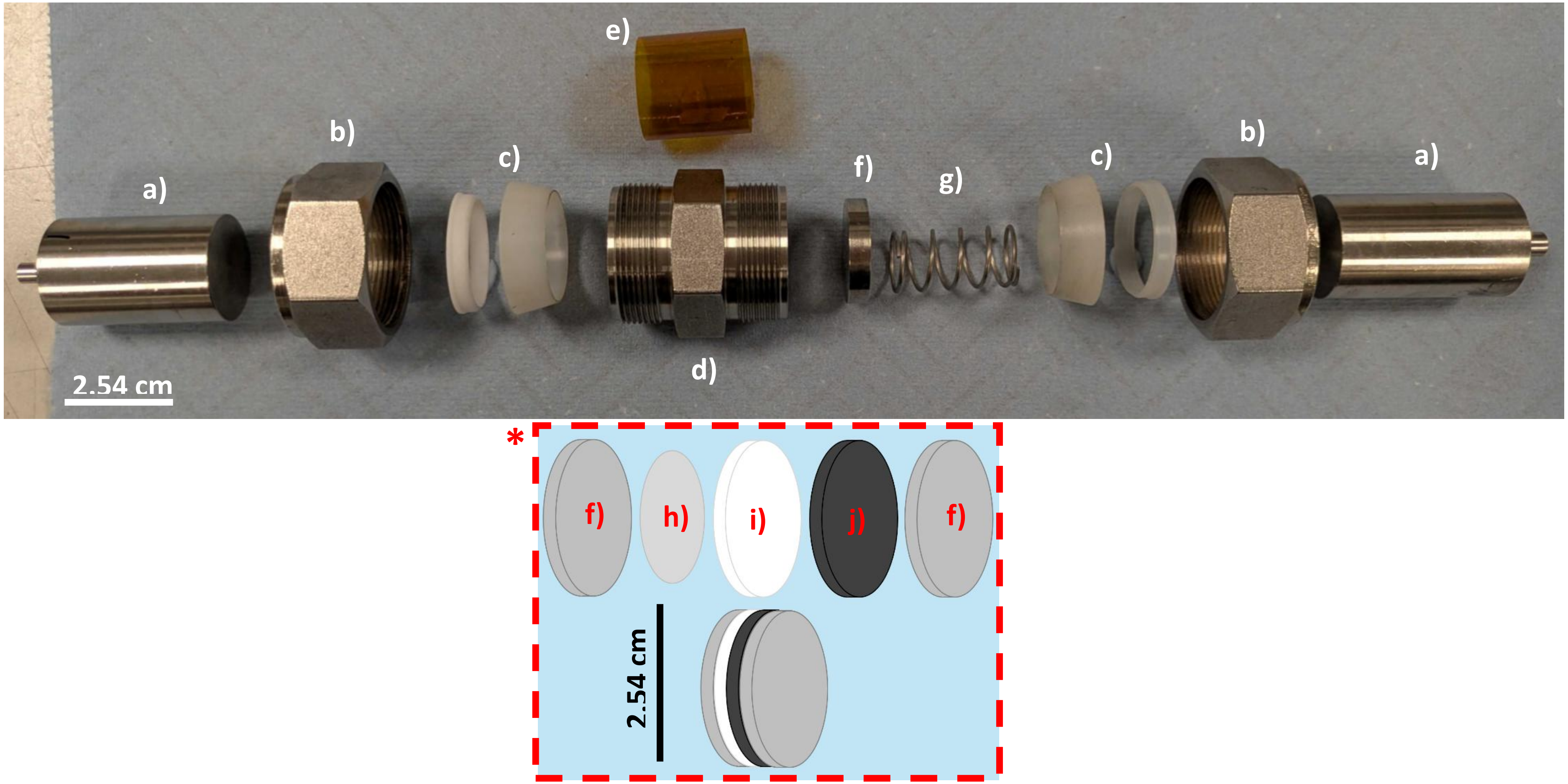

Figure S2: Large 1" diameter Swagelok cell components. A) Stainless steel plunger, b) outer cell body screw, c) PTFE O-ring and seal, d) stainless steel cell main body, e) Kapton film (internal to cell body during operation), f) stainless steel spacer/current collector, g) stainless steel spring. *Internal cell body additionally contains h) Na metal anode i) two glass fibre separators with 1M $NaPF_6$ electrolyte j) $NaNiO_2$ powder/carbon mixture cathode. Note that the cell is formed between either two stainless steel spacers/current collectors, or a spacer and plunger, depending on size of springs available.*

S-3: Confirmation of Sample Identities & Phase Purity

The pristine active cathode material ($NaNiO_2$, $O'3$) was synthesised as described in Methods. To confirm the identity and phase purity of the sample, Rietveld refinement of the literature reported structure against SXRD data was performed (Figure S12, R_{wp} = 7.97 %).[5] Due to exposure to air and moisture a minor de-sodiated phase impurity (1.86[12] wt%) was identified from its corresponding (001) reflection (~1.167 $Å^{-1}$), and was modelled by the pristine $O'3$, with lattice parameters allowed to refine to reflect the increase in interlayer spacing (1.201 $Å^{-1}$ in pristine $O'3$). Whilst this will not reflect the precise $O''''3$ structure, the model is suitable to capture the only visible reflection (001) in the measured pattern.

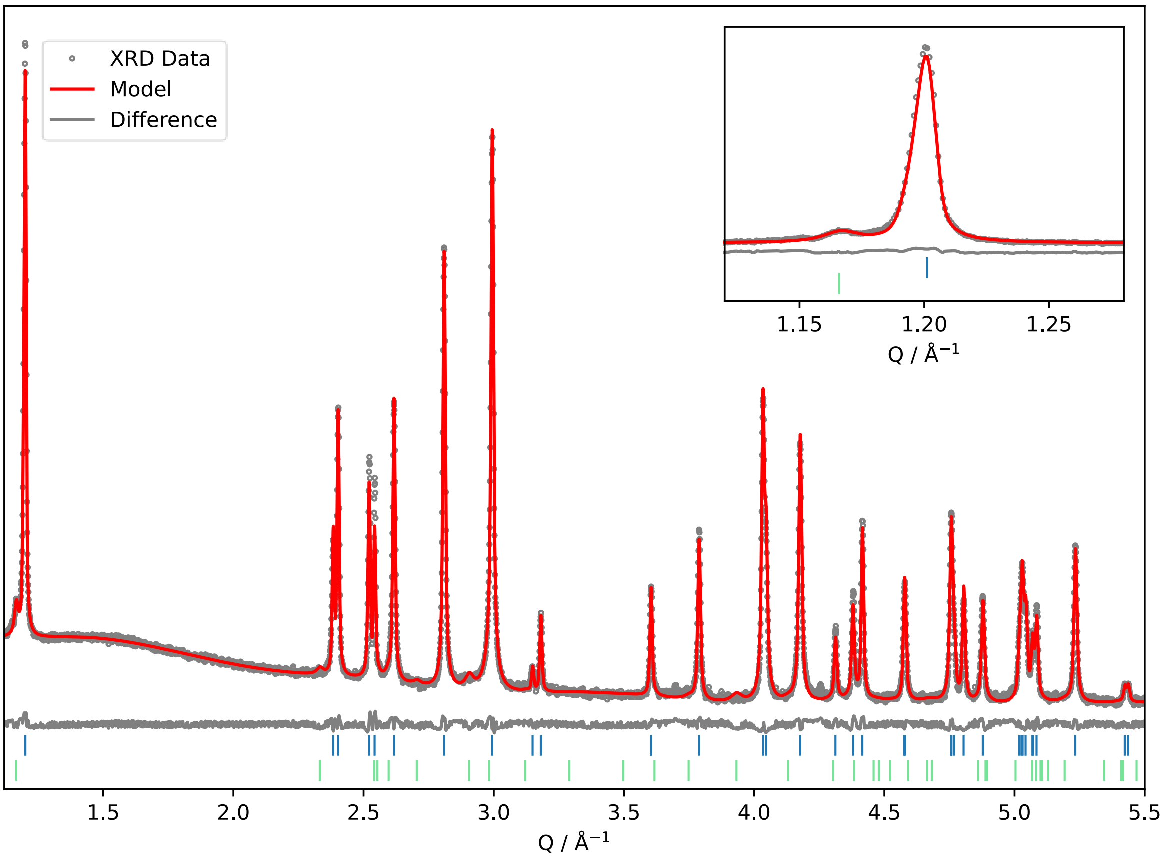

Figure S3: Rietveld refinement of SXRD for $NaNiO_2$. Y-axis is square root of intensity for clarity. Tick marks show $O'3$ (blue) and a minor $O''''3$ impurity (green). Inset displays (001) reflection region expanded for visual clarity.

For the *ex situ* samples, phase purity should occur exactly at the voltage step mid-point associated with the phase transition (Figure S3). The Swagelok cells have variable electrochemistry, owed to the inconsistent pressure applied by the spring/manual tightening of the steel plungers. As such, there is an observable discrepancy between the voltage curves of the two cells (Figures SI4a-b). Nonetheless, through producing the $P'3$ *ex situ* sample *via* Swagelok route, as described in methods, a highly phase pure sample was obtained (Figure S4a-d). The Swagelok sample stopping capacity corresponds to 77.71 $mAh.g^{-1}$, equivalent to 0.330 Na^+ removed per formula unit, giving $Na_{0.670}NiO_2$ ~ $Na_{2/3}NiO_2$.

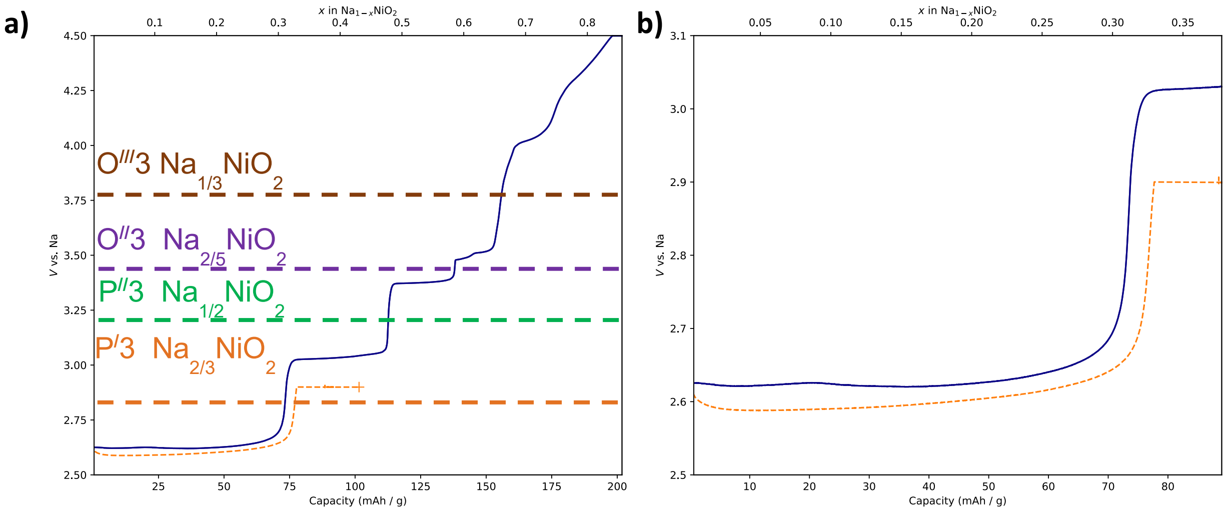

Figure S4: Electrochemical charge profile of $NaNiO_2$ cells (purple), with charge to desired voltage for $P^{/}3$ phase overlayed (orange). a) Swagelok cell (C/100, 24h voltage hold), b) expanded plot of ex-situ ample charge region for visual clarity.

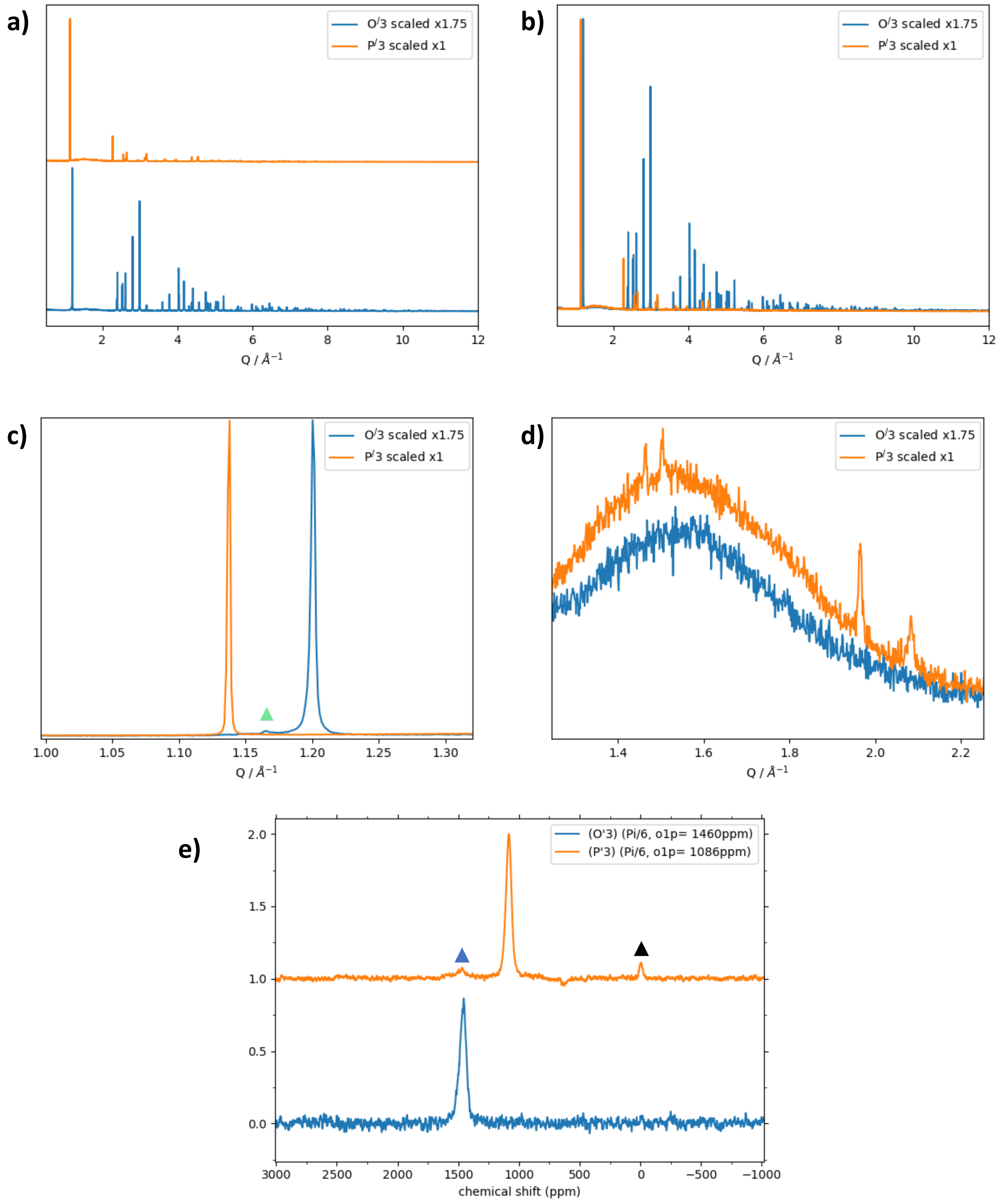

Figure S5: a-d) SXRD patterns and e) ^{23}Na ssNMR pj-MATPASS (60 kHz) spectra of the pristine $O^{\prime}3$ cathode powder (blue) and $P^{\prime}3$ ex situ sample (orange), a) offset and b) overlayed for clarity. c) The layered (001) peak region has been expanded to demonstrate presence of a minor impurity phase fraction of $O^{\prime\prime\prime\prime}3$ (green) in $O^{\prime}3$ (blue). d) Superstructure peaks in the $P^{\prime}3$ sample. Environments from the $O^{\prime}3$ impurity (blue) and a diamagnetic impurity (black) are present in the $P^{\prime}3$ sample.

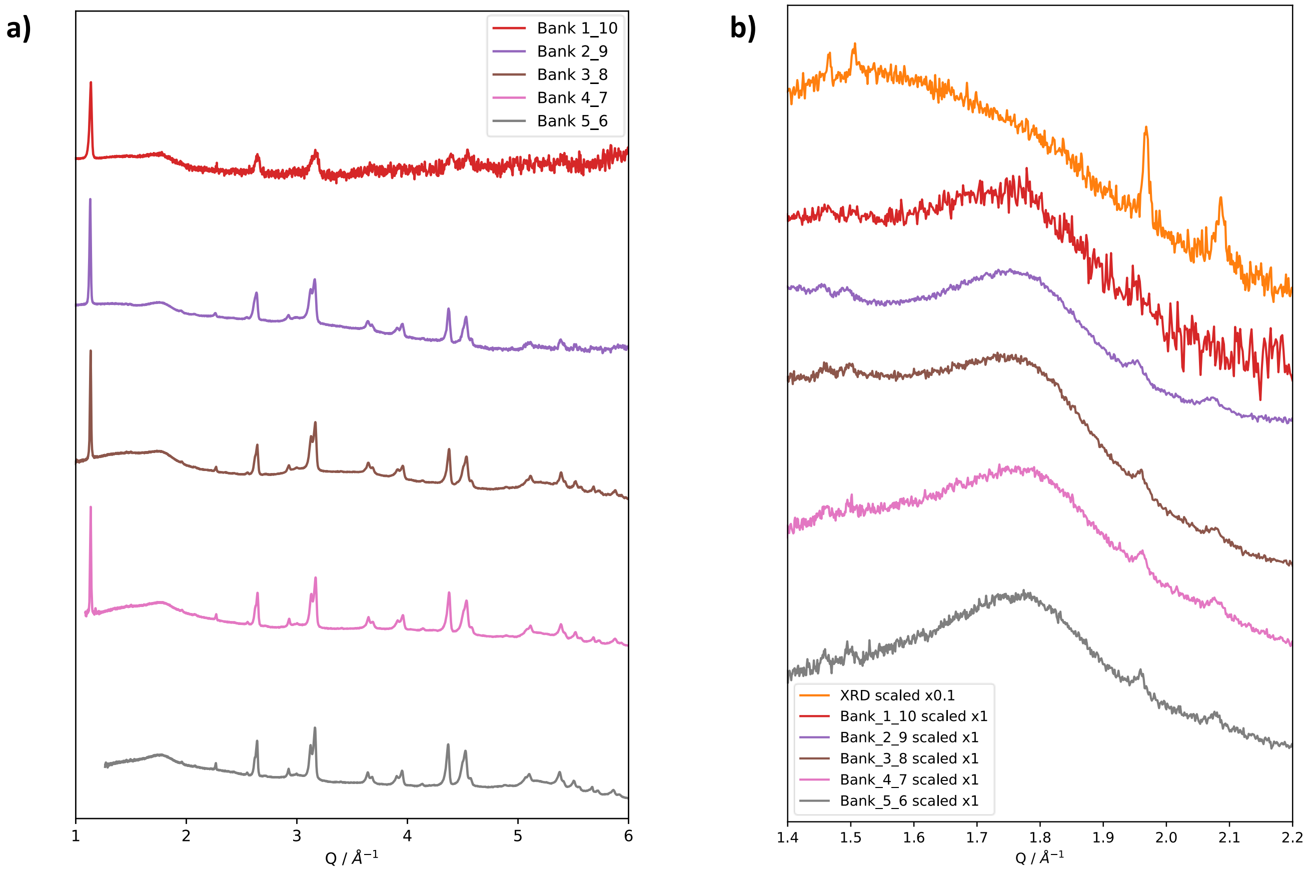

Figure S6: a) NPD patterns of the P/3 ex situ sample from the 5 pairs of equivalent banks (i.e. 1 and 10 are combined) of the WISH diffractometer (average 2θ of bank pairs 1_10 = 27.0 °, 2_9 = 58.33 °, 3_8 = 90.00 °, 4_7 = 121.66 °, 5_6 = 152.82 °). b) Superstructure peaks in the P/3 sample are displayed, with SXRD data provided for reference (orange). Intensity is presented on a square root scale for visual clarity.

S-4: Initial Structural Model for $P^{/}3$

We utilised the ISODISTORT online software package to produce a starting structural model for the $P^{/}3$ phase. Using a prismatic proxy of $NaNiO_2$ (monoclinic cell choice 2) as the starting point, we then incorporated a 3-fold expansion of cell in the b-lattice direction using the irreducible representation LD2 k2t2 with order parameter direction P1. The cell parameters are very similar to those reported by de Boisse *et al.* ($cell_{super}$, space group = $P2_1/a$, a_{super} = 4.972 Å, b_{super} = 8.589 [$3*b_{O'3}$] Å, c_{super} = 5.739 Å, β_{super} = 105.84 °), where a and c are swapped due to the different chosen cell setting.[6] Our identified *$P2_1/c$* cell setting maintains crystallographic convention of using the standard cell setting. The alternative cell setting reported by de Boisse allows for interlayer distance = *c* sin*(*β*),* whilst in our standard crystallographic cell this would be *a* sin*(*β*)* .[6,7]

Table ST1: Structural parameters for the prismatic proxy cell starting point cell for $Na_{2/3}NiO_2$.

**Space group**	***a* (Å)**	***b* (Å)**	***c* (Å)**	**β (°)**	**Unit-cell volume (Å^3)**
C2/m	5.749263	2.861633	4.971325	105.975731	78.630783
**Element**	**Wykoff position**	**x**	**y**	**z**	**Occupancy**
Ni1	2a	0	0	0	1
Na1	4i	0.500000	0	0.775000	0.667
O1	4i	0.843900	0	0.622000	1

Table ST2: Symmetry allowed cell parameters generated from (1x3x1) expansion of parent starting cell using ISODISTORT, and results of Pawley fits to the SXRD data. Here, "Additional DoF" represents extra allowed reflections that are not present in the data, but are allowed by symmetry of the cell, in the superstructure peak region (Q =). "GoF" represents the R_{wp} of the fit, multiplied by the number of additional DoFs for a cell, and then divided by the maximum number of possible additional DoFs, to give a balanced fit parameter which accounts both for the R_{wp}, and the number of spurious reflections.

**Space group**	**IR**	**OPD**	***a (Å)***	***b (Å)***	***c (Å)***	**β (°)**	**Unit-cell volume (Å^3)**	**R_{wp}**	**Additional DoFs**	**GoF**
P2	*LD1 k2t1*	C1	4.97395(20)	8.57713(33)	5.75098(6)	106.10797(23)	235.717(14)	9.447	8	9.45
P2/m		P1	4.97350(15)	8.58193(29)	5.74916(6)	106.04478(21)	235.828(11)	8.942	8	8.94
P2/c		P2	5.75170(6)	8.57520(33)	4.97366(19)	106.13295(24)	235.650(13)	9.739	5	6.09
$P2_1$	***LD2 k2t2***	C1	4.97389(19)	8.57599(33)	5.75150(6)	106.12596(24)	235.683(14)	9.607	7	8.41
$P2_1/c$		**P1**	5.74396(8)	8.58869(18)	4.97058(14)	105.89028(27)	235.844(9)	6.042	**4**	3.02
$P2_1/m$		P2	4.97297(20)	8.58157(26)	5.74960(6)	106.05984(23)	235.793(12)	9.093	7	7.96

Table ST3: Structural parameters for the refinement starting point cell for $Na_{2/3}NiO_2$ generated by from IR LD2 k2t2.

**Space group**	***a* (Å)**	***b* (Å)**	***c* (Å)**	**β (°)**	**Unit-cell volume (Å^3)**
$P2_1/c$	5.749260	8.584900	4.971330	105.975731	235.892499
**Element**	**Wykoff position**	***x***	***y***	***z***	**Occupancy**
Ni1_1	2a	0	0	0	1
Ni1_2	4e	0.000000	0.166670	0.500000	1
Na1_1	4e	0.500000	0.000000	0.745000	0.667
Na1_2	4e	0.500000	0.166670	0.245000	0.667
Na1_3	4e	0.500000	0.666670	0.745000	0.667
O1_1	4e	0.843900	0.000000	0.622000	1
O1_2	4e	0.843900	0.166670	0.122000	1
O1_3	4e	0.843900	0.666670	0.622000	1

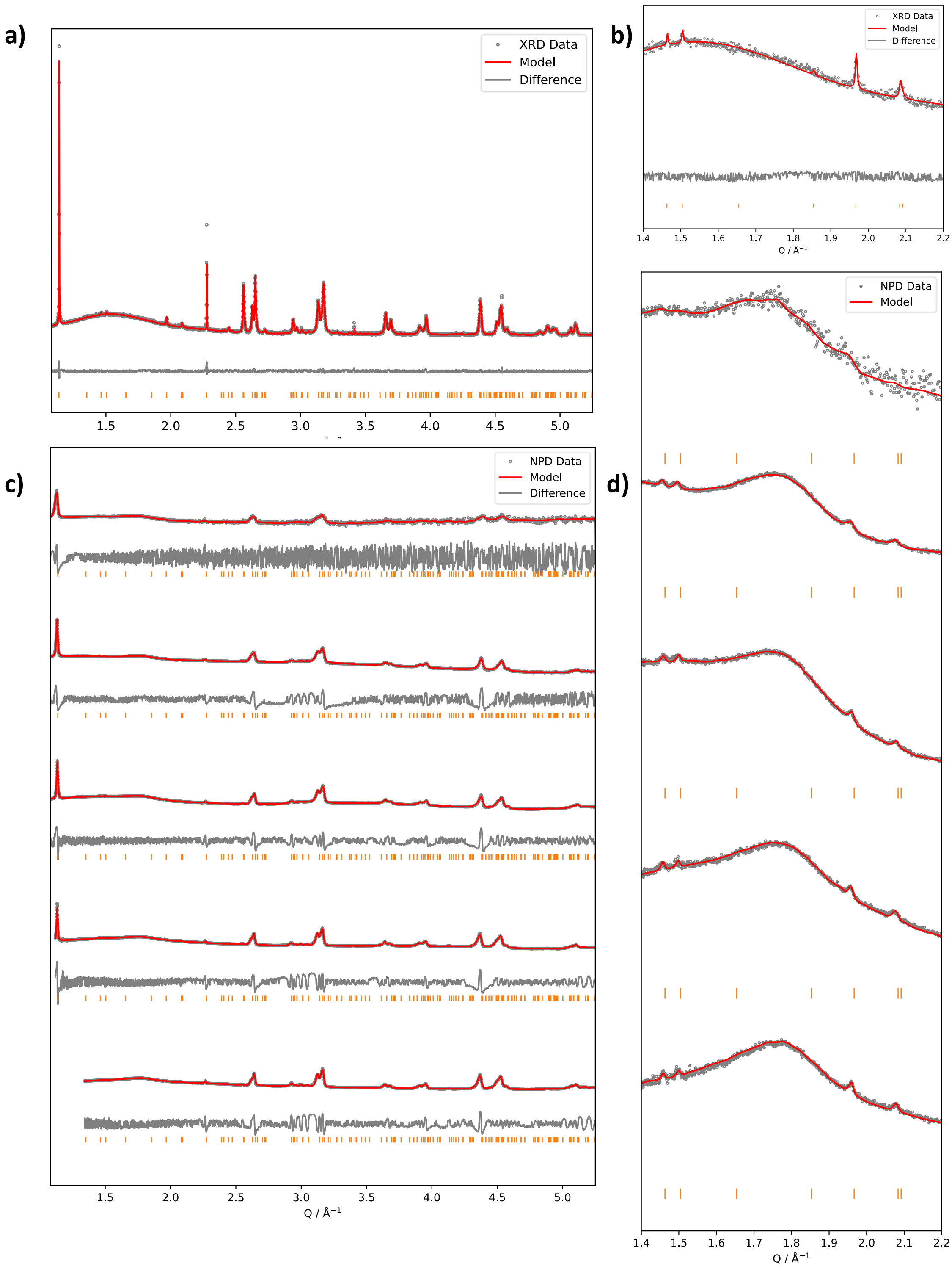

Figure S7: Combined Pawley fitting of reported cell parameters (cell$_{super}$, space group = $P2_1/a$, a_{super} = 4.972 Å, b_{super} = 8.589 [3~$b_{O'3}$] Å, c_{super} = 5.739 Å, β_{super} = 105.84 °), to a) SXRD and b) NPD data.[6] The calculated patterns for each of the NPD banks are presented from bank pair 1_10 (top) to bank 5_6 (bottom) (average 2θ of bank pairs 1_10 = 27.0 °, 2_9 = 58.33 °, 3_8 = 90.00 °, 4_7 = 121.66 °, 5_6 = 152.82 °). Y-axis is square root of intensity for visual clarity. Superstructure peak region in the b) SXRD and c) NPD data, expanded for visual clarity.*

S-5: Bond Valence Sum (BVS) Method Calculations

The bond valence sum method has been applied to approximate Ni charge states, as per Equation SE1 wherein V is the total bond valence sum, v_i is the bond valence contribution from each bond, R_i is the observed (calculated via Rietveld refinement) bond length, R_0 is the tabulated ideal bond length, and B_0 is an empirical constant.[8]

$$\boldsymbol{V = \sum_{i=1}^{6}(v_i)}$$

$$\boldsymbol{v_i = exp\left(\frac{R_0 - R_i}{B_0}\right)} \qquad \text{(Eq. SE1)}$$

Ni in $NaNiO_2$ should, nominally, exist as Ni^{3+} in an entirely ionic system. Due to the non-uniform bond length distribution of the Ni-O bonds in $NaNiO_2$, we initially calculated the BVS for Ni in $NaNiO_2$, using both the ideal bond length (R_0) and empirical constant (B_0) values for Ni^{3+}-O^{2-} ($R_{0,Ni3+}$, $B_{0,Ni3+}$), and Ni^{4+}-O^{2-} ($R_{0,Ni4+}$, $B_{0,Ni4+}$) bonds (1.75, 0.37 Å and 1.734, 0.335 Å respectively).[9] Using $R_{0,Ni3+}$ results in $Ni^{3.29+}$. Using $R_{0,Ni4+}$ results in $Ni^{2.95+}$, which is significantly closer to the expected oxidation state of Ni^{3+}. Therefore $R_{0,Ni4+}$ was used to calculate Ni charge states for the $Na_{2/3}NiO_2$ phase, resulting in an average value of $Ni^{3.31+}$ as described in the main text.

S-6: Details of the $Na_{2/3}NiO_2$ Combined Refinement

Table ST4: Structural parameters for $Na_{2/3}NiO_2$ as determined from combined Rietveld refinement of SXRD and NPD data (R_{wp} = 1.473, χ^2 = 1.444)

**Space group**	**a (Å)**	**b (Å)**	**c (Å)**	**β (°)**	**Unit-cell volume (Å^3)**	
$P2_1/c$	5.74466(3)	8.58914(11)	4.97153(7)	105.9132(10)	235.903(10)	
**Element**	**Wykoff position**	**x**	**y**	**z**	**Occupancy**	**B_{iso} (Å^2)**
Ni1	2a	0	0	0	1	0.88(8)
Ni2	4e	-0.0079(6)	-0.6635(5)	0.9995(8)	1	1.15(5)
Na	4e	0.489(1)	0.6647(15)	0.8021(17)	1	*
O1	4e	0.809(2)	-0.508(2)	0.090(3)	1	0.500(10)
O3	4e	0.833(2)	0.168(3)	0.112(3)	1	0.78(10)
O2a	4e	-0.848(3)	-0.690(2)	1.373(5)	0.5	0.65(14)
O2b	4e	-0.779(3)	-0.660(2)	0.374(5)	0.5	0.65(14)
*** Na B_{iso}**	**U_{11} (Å^2)**	**U_{22} (Å^2)**	**U_{33} (Å^2)**	**U_{12} (Å^2)**	**U_{13} (Å^2)**	**U_{23} (Å^2)**
	0.029(3)	0.103(7)	0.033(5)	0.007(13)	0.009(4)	-0.052(9)

Table ST5: Structural parameters for $Na_{2/3}NiO_2$ as determined from geometry optimisation of the combined refinement cell. Note that goodness of fit parameters are given based on the fit of the below unrefined cell against the data. No error values or B_{iso} values are given below as the cell is not refined against the data. (R_{wp} = 2.855, χ^2 = 2.795)

**Space group**	**a (Å)**	**b (Å)**	**c (Å)**	**β (°)**	**Unit-cell volume (Å³)**
Pc	5.73563	8.74812	4.91403	105.2960	237.8324
**Element**	**Wykoff position**	**x**	**y**	**z**	**Occupancy**
Ni1	2a	0.998634	0.251004	0.499290	1
Ni2A	2a	0.984737	0.083765	0.001434	1
Ni2B	2a	0.013728	0.418353	0.001202	1
Na1	2a	0.494721	0.387867	0.791662	1
Na2	2a	0.505277	0.108009	0.203115	1
O1	2a	0.170752	0.094368	0.381253	1
O2	2a	0.175771	0.250793	0.889774	1
O3	2a	0.201248	0.432688	0.375111	1
O4	2a	0.800584	0.074084	0.623478	1
O5	2a	0.816651	0.272657	0.117530	1
O6	2a	0.835684	0.420014	0.615634	1

Table ST6: Structural parameters for $Na_{2/3}NiO_2$ as determined from combined geometry optimisation of the AIMD snapshot "SD" cell. Note that goodness of fit parameters are given based on the fit of the below unrefined cell against the data. No error values or B_{iso} values are given below as the cell is not refined against the data. (R_{wp} = 2.905, χ^2 = 2.843)

**Space group**	**a (Å)**	**b (Å)**	**c (Å)**	**β (°)**	**Unit-cell volume (Å³)**
$P2_1/c$	5.74735	8.63768	4.99574	105.7831	238.6572
**Element**	**Wykoff position**	**x**	**y**	**z**	**Occupancy**
Ni1	2a	0.000000	0.000000	0.500000	1
Ni2	4e	0.006432	0.166490	0.004061	1
Na1	4e	0.490366	0.143155	0.800889	1
O1	4e	0.176453	0.679579	0.874343	1
O2	4e	0.183080	0.501482	0.413590	1
O3	4e	0.189980	0.179779	0.383476	1

*Table ST7: Bond lengths and average BVS values for the $Ni_{(1)}$ and $Ni_{(2)}$ sites, and average Ni BVS in the proposed split O-site average structural model. Bond lengths are colour coded across all values, wherein darkest red are the shortest, and darkest green are the longest. The */^ symbols in the average split O-site models represent the average short (*) and average long (^) Ni-O bond values for the split site, as there are two values for long and short in the $Ni_{(2)}$ site.*

	$Ni_{(1)}$	$Ni_{(2)}$
	1.824(9)	1.853(9)
	1.824(9)	1.88(6)*
	1.9140(26)	1.893(5)
	2.094(5)	1.897(28)
	2.094(5)	1.99(5)^
	2.172(24)	2.063(8)
Average BVS	3.07(2)	3.43(2)
	3.31(1)	

Table ST8: Bond lengths and average BVS values for the $Ni_{(1)}$, $Ni_{(2A)}$ and $Ni_{(2B)}$ sites, and average Ni BVS in Jahn-Teller Distorted (DFT-JT) model. Bond lengths are colour coded across all values, wherein darkest red are the shortest, and darkest green are the longest.

	$Ni_{(1)}$	$Ni_{(2A)}$	$Ni_{(2B)}$
	1.872	1.881	1.868
	1.889	1.892	1.900
	1.892	1.928	1.915
	1.895	1.988	1.920
	1.901	2.061	2.103
	1.913	2.068	2.150
Average BVS	3.73	3.04	3.06
	3.27		

Table ST9: Bond lengths and average BVS values for the $Ni_{(1)}$ and $Ni_{(2)}$ sites, and average Ni BVS in geometry optimised AIMD Spin Disproportionated (SD) model. Bond lengths are colour coded across all values, wherein darkest red are the shortest, and darkest green are the longest.

	**$Ni_{(1)}$**	**$Ni_{(2)}$**
	2.043	1.893
	2.043	1.894
	2.048	1.895
	2.048	1.900
	2.071	1.905
	2.071	1.906
Average BVS	2.31	3.67
	3.22	

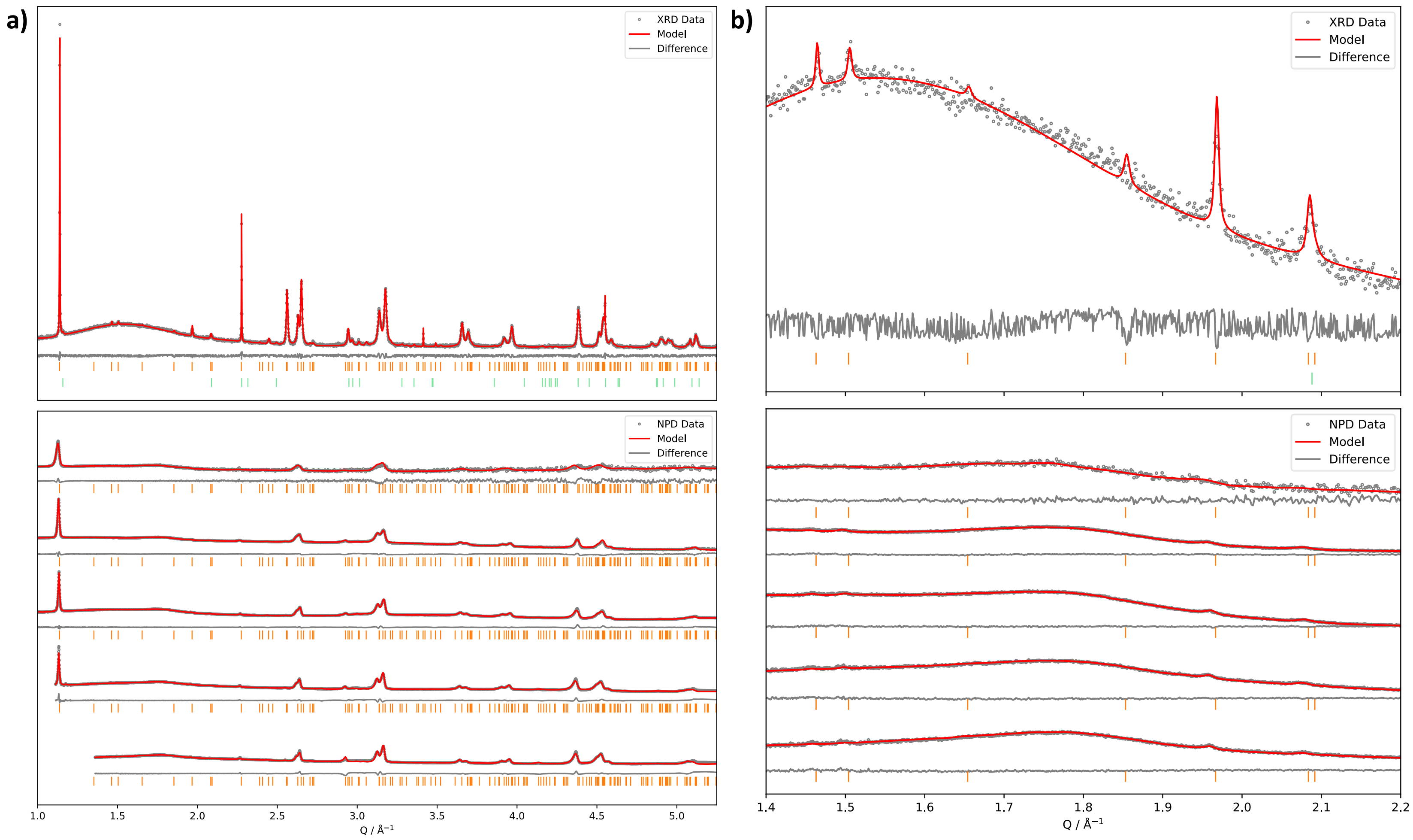

Figure S8: a) Combined refinement of SXRD (top) and NPD (bottom). Y-axis plotted as square root of intensity for visual clarity. The SXRD presents a minor $O^{////}D$ impurity (green tick marks). The calculated patterns for each of the NPD banks are presented with from bank 1 (top), to bank 5 (bottom) (average 2θ of bank pairs 1_10 = 27.0 °, 2_9 = 58.33 °, 3_8 = 90.00 °, 4_7 = 121.66 °, 5_6 = 152.82 °). Superstructure peak region in the b) SXRD and c) NPD data, expanded for visual clarity.

S-7: Magnetic Characterisation and Curie-Weiss Fitting

The DC susceptibility data (Figure S9a) shows paramagnetic behaviour above ~25 K, with an apparent magnetic transition at this temperature indicating the presence of magnetic ordering or glassiness at low temperature. The magnetic susceptibility was calculated in the low field limit ($\chi = \frac{dM}{dH} \approx \frac{M}{H}$). At temperatures well above the ordering temperature, the magnetic susceptibility (*χ)* can be fit to the Curie-Weiss law (Figure S9a):

$$\chi = \frac{C}{T - \theta} \qquad \text{(Eq. SE2)}$$

With Curie Constant (C) = 0.251(1) emu K mol^{-1}, Weiss temperature (θ_{CW}) = - 0.2(7) K, and χ_0 = -5.5(3) x 10^{-5} emu mol^{-1} Oe^{-1}. This Curie constant corresponds to an effective magnetic moment (μ_{eff}) of 1.416(4) μ_B, which is in between the expected moments for Ni^{3+} (S=½, μ_{eff}= 1.73 μ_B) and Ni^{4+} (S=0, μ_{eff}= 0 μ_B).

We can compare these results to our structural model, which contains two Ni sites with relative multiplicity 1:2. The bond-valance sum for the minority site indicates it is close to a nominal Ni^{3+} state, while the second site is close to $Ni^{3.5+}$. The expected observed magnetic moment (per Ni ion) for such an ensemble is given by Equation SE3:

$$\mu_{\text{eff}} = \sqrt{c_1 \mu_1^2 + c_2 \mu_2^2} \qquad \text{(Eq. SE3)}$$

Where c_1 and c_2 are the fractional concentrations of each ion and μ_1 and μ_2 are the effective magnetic moments for each state. Assuming a simple hypothetical model where $Ni_{(1)}$ is Ni^{3+} (with one unpaired e^-), whilst one unpaired e^-hops between $Ni_{(2)}$ sites, giving a mixture of $Ni^{3+/4+}$ sites:

$$\mu_{\text{eff}} = \sqrt{\frac{1}{3}\mu^2_{Ni^{3+}_{(1)}} + \frac{1}{3}\mu^2_{Ni^{3+}_{(2)}} + \frac{1}{3}\mu^2_{Ni^{4+}_{(2)}}} = \mathbf{1.413\ \mu_B}$$

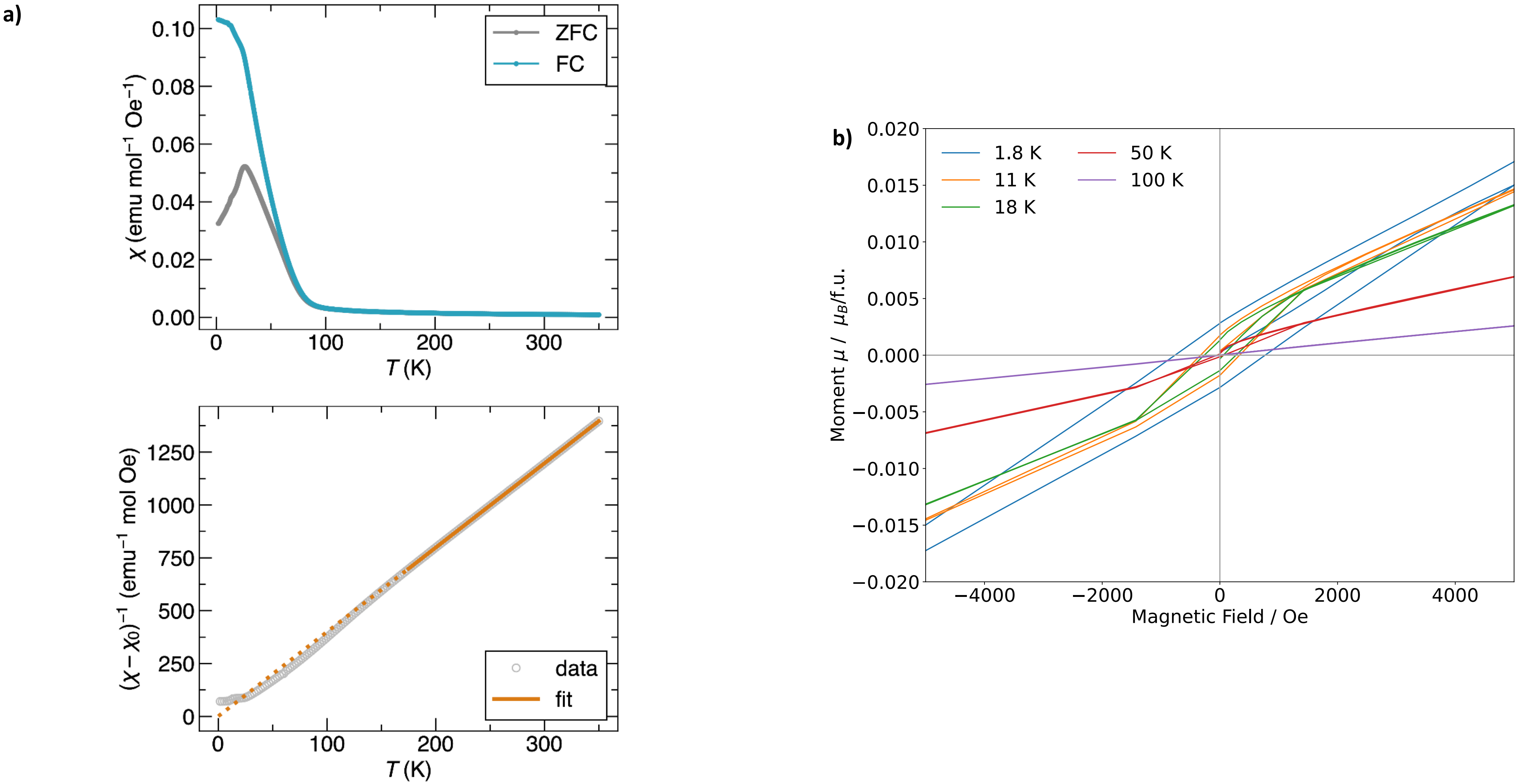

Figure S9: a) (Top) DC magnetic susceptibility data from 1.8 – 350 K measured on heating at 100 Oe, cooled under zero field (blue) and under 100 Oe field (red). (Bottom) Curie-Weiss fitting of FC inverse DC magnetic susceptibility from 175 – 350 K collected under a field of 20 kOe. Data points (grey circles), fit (blue dotted line). b) Magnetisation vs. field with varying temperature from 1.8 K to 100 K for $Na_{2/3}NiO_2$.

S-8: Ni-O Coordination Environments and Ni Spins in the Structural Models

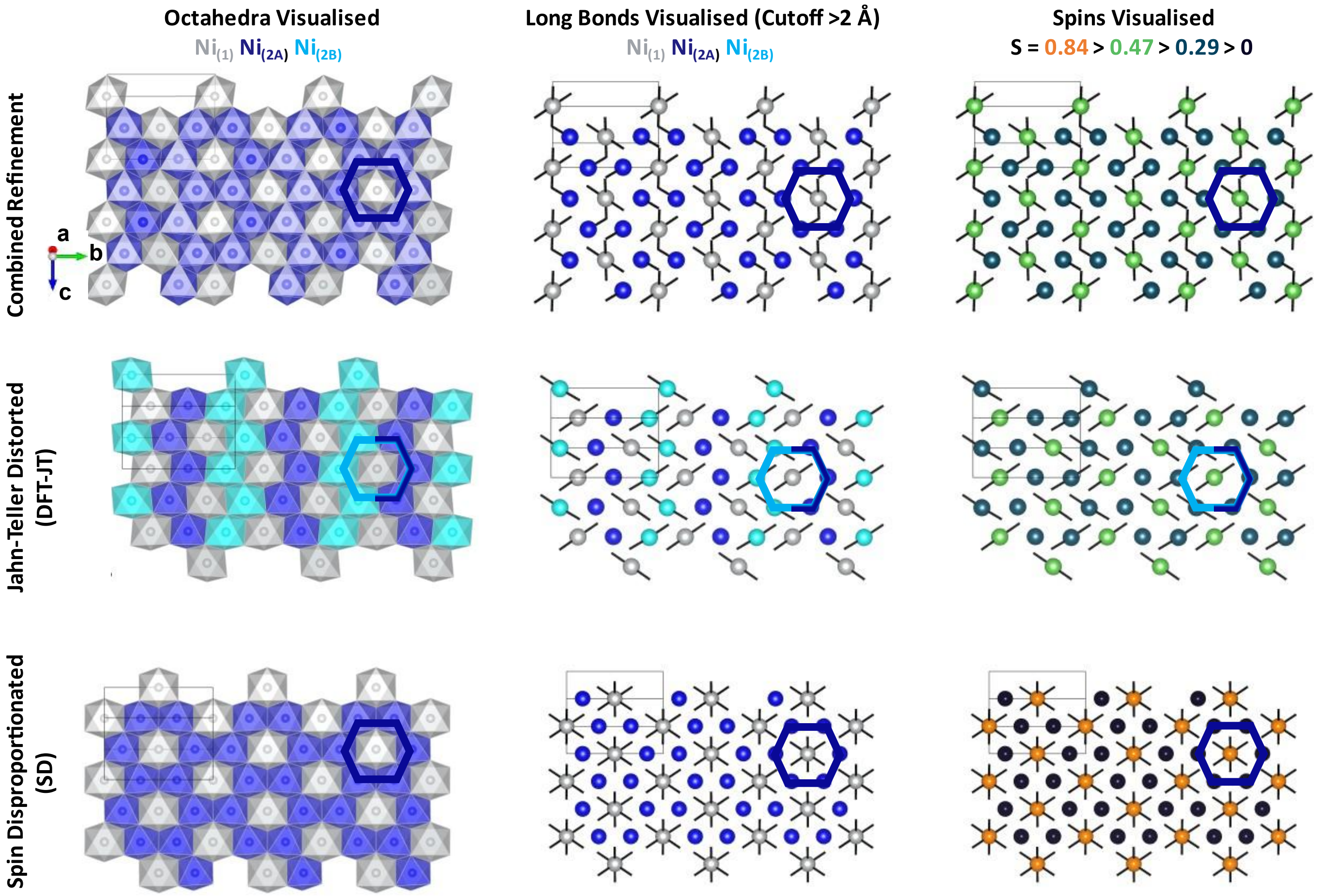

Figure S10: Combined refinement (top row), geometry optimised combined refinement "Jahn-Teller Distorted (DFT-JT)" (middle row), and geometry optimised AIMD "Spin Disproportionated (SD)" (bottom row) structures. Visualisations are: octahedra colour coded by crystallographic site (first column), atomic structure colour coded by crystallographic site (second column), atomic structure colour coded by calculated spin (third column). All bonds drawn with cutoff >2.0 Å. In the "ball and stick" style figures, atoms have been enlarged by comparison to the octahedral figures for visual clarity.

S-9: A van Vleck Analysis of AIMD Trajectories

The AIMD trajectories with increasing temperature, provided insight into the suggested displacive nature of the material. At 25 K, there were demonstrably two octahedral volumes (S11). With increasing temperature, the two discrete octahedral volumes, and Ni-O bond lengths, merge into a continuum. The undistorted nature of the two types of octahedra was demonstrated by the single spot at the pole of the Q_2/Q_3 mode plot, showing that the Ni-O bond lengths are uniform (S12). On increasing temperature, the distribution of octahedral volumes is still greatest at the pole, but there are clearly octahedra which are distorted preferentially along the 3 axes of elongation. The lack of 3 discrete sites of high intensity in these plots demonstrates the displacive/dynamic nature of the material, as opposed to an order-disorder case. We have previously reported this behaviour with increasing temperature in the related (fully sodiated) $NaNiO_2$ material.[10] Note that it may visually appear that the $Ni_{(2)}$ site has greater probability at the extremes of the plot, than the $Ni_{(1)}$ site. However, we assert that this is likely due to the presence of twice as many $Ni_{(2)}$ sites as $Ni_{(1)}$ sites in the model, naturally leading to more dense occupation throughout the entire plot, as it visually apparent.

For further explanation of the van Vleck plots in Figure S12, see Nagle-Cocco *et al.*[10]

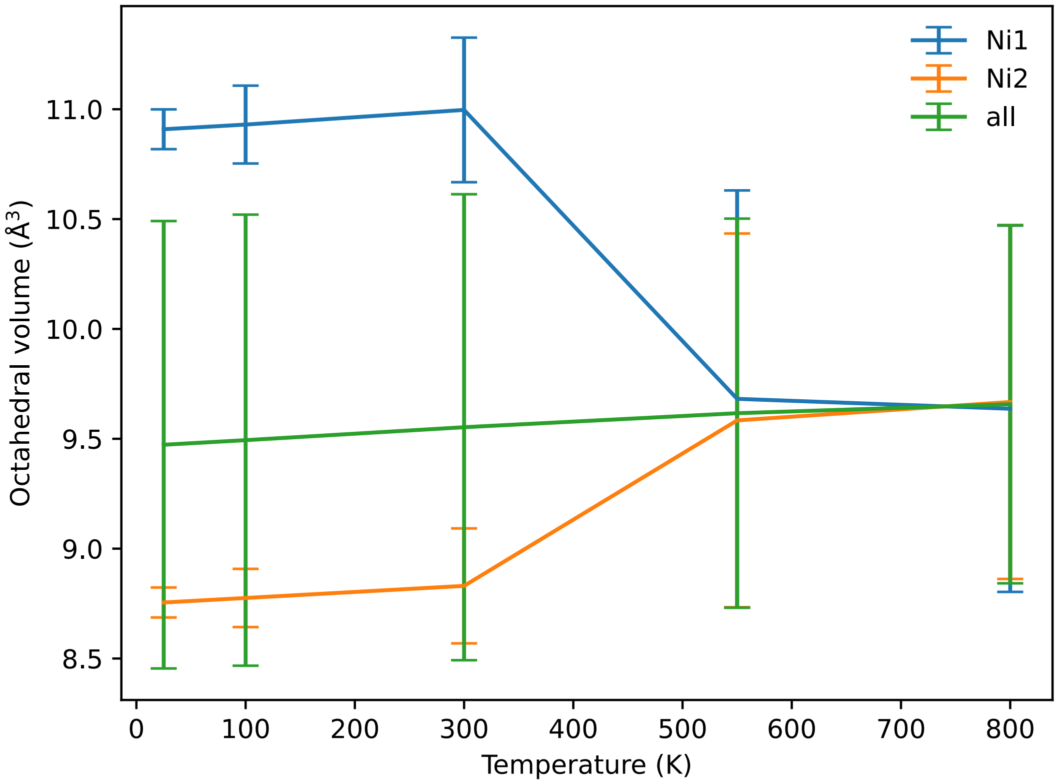

Figure S11: Octahedral volumes as calculated by VanVleckCalculator for each Ni site present in snapshots of the AIMD trajectories as a function of temperature from 25 – 800 K.

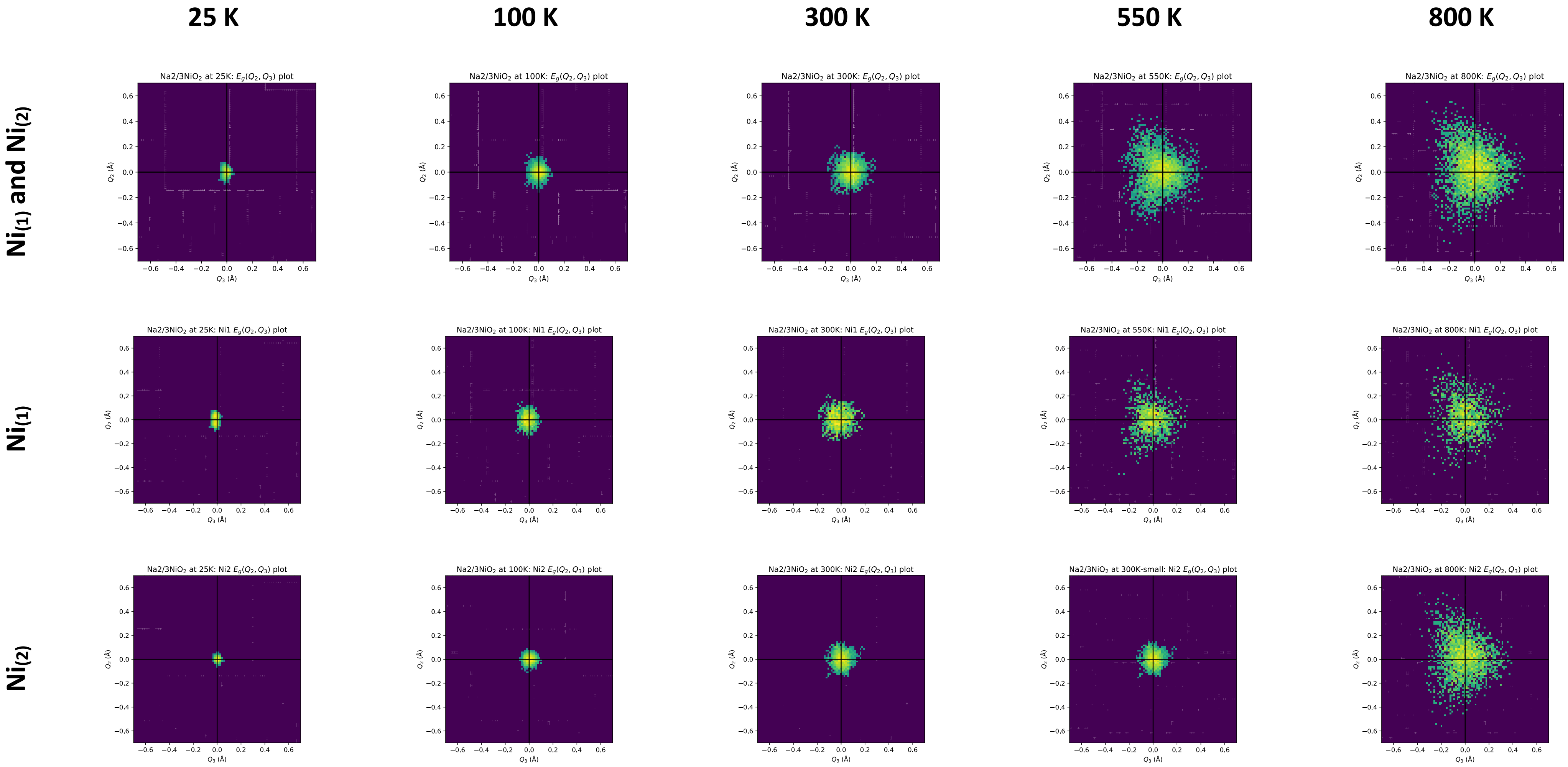

Figure S12: The van Vleck analysis of the Q_2/Q_3 modes present in AIMD trajectories with increasing temperature from 25 – 800 K, representing both Ni sites (top), $Ni_{(1)}$ sites (middle), and $Ni_{(2)}$ sites (bottom).

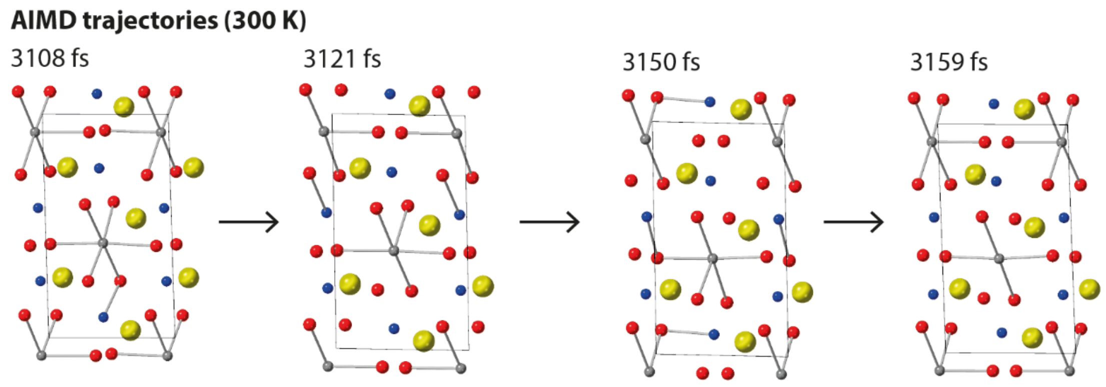

Figure S13: Snapshots from AIMD trajectories at 300 K showing fluctuations in the local Ni-O coordination. Bonds with lengths >2 Å are drawn.

References:

(1) Takahashi, Y.; Gotoh, Y.; Akimoto, J. Single-Crystal Growth, Crystal and Electronic Structure of $NaCoO_2$. *J. Solid State Chem.* **2003**, *172* (1), 22–26. https://doi.org/10.1016/S0022-4596(02)00042-7.

(2) Kubota, K.; Asari, T.; Yoshida, H.; Yaabuuchi, N.; Shiiba, H.; Nakayama, M.; Komaba, S. Understanding the Structural Evolution and Redox Mechanism of a $NaFeO_2$–$NaCoO_2$ Solid Solution for Sodium-Ion Batteries. *Adv. Funct. Mater.* **2016**, *26* (33), 6047–6059. https://doi.org/10.1002/adfm.201601292.

(3) de Brion, S.; Darie, C.; Holzapfel, M.; Talbayev, D.; Mihály, L.; Simon, F.; Jánossy, A.; Chouteau, G. Spin Excitations in the Antiferromagnet $NaNiO_2$. *Phys. Rev. B* **2007**, *75* (9), 094402. https://doi.org/10.1103/PhysRevB.75.094402.

(4) Nagle-Cocco, L. A. V.; Bull, C. L.; Ridley, C. J.; Dutton, S. E. Pressure Tuning the Jahn–Teller Transition Temperature in $NaNiO_2$. *Inorg. Chem.* **2022**, *61* (10), 4312–4321. https://doi.org/10.1021/acs.inorgchem.1c03345.

(5) Holzapfel, M.; Darie, C.; Bordet, P.; Chappel, E.; Núñez-Regueiro, M.-D.; Diaz, S.; de Brion, S.; Chouteau, G.; Strobel, P. Mixed Layered Oxide Phases $NaxLi_{1-x}NiO_2$: A Detailed Description of Their Preparation and Structural and Magnetic Identification. *Solid State Sci.* **2005**, *7* (5), 497–506. https://doi.org/10.1016/j.solidstatesciences.2004.10.046.

(6) de Boisse, B. M. Structural and Electrochemical studies of $NaxMn_{1-y}Fe_yO_2$ and $NaNiO_2$ materials as positive electrode for Na-ion batteries. 283.

(7) Stokes, H. T.; Hatch, D. M.; Campbell, B. J. *ISODISTORT*. ISODISTORT, ISOTROPY Software Suite. iso.byu.edu.

(8) Brown, I. D. Recent Developments in the Methods and Applications of the Bond Valence Model. *Chem. Rev.* **2009**, *109* (12), 6858–6919. https://doi.org/10.1021/cr900053k.

(9) *Bond valence parameters*. https://www.iucr.org/resources/data/datasets/bond-valence-parameters (accessed 2024-11-04).

(10) Nagle-Cocco, L. A. V.; Genreith-Schriever, A. R.; Steele, J. M. A.; Tacconis, C.; Bocarsly, J. D.; Mathon, O.; Neuefeind, J. C.; Liu, J.; O'Keefe, C. A.; Goodwin, A. L.; Grey, C. P.; Evans, J. S. O.; Dutton, S. E. Displacive Jahn–Teller Transition in $NaNiO_2$. *J. Am. Chem. Soc.* **2024**. https://doi.org/10.1021/jacs.4c09922.
